# Unique Method for Facile Postsynthetic Modification of Nonisocyanate Polyurethanes

**DOI:** 10.1021/acs.macromol.3c02232

**Published:** 2024-02-29

**Authors:** Sergei V. Zubkevich, Maksim Makarov, Reiner Dieden, Laura Puchot, Vincent Berthé, Stephan Westermann, Alexander S. Shaplov, Daniel F. Schmidt

**Affiliations:** Luxembourg Institute of Science and Technology (LIST), 5, Avenue des Hauts-Fourneaux, L-4362 Esch-sur-Alzette, Luxembourg

## Abstract

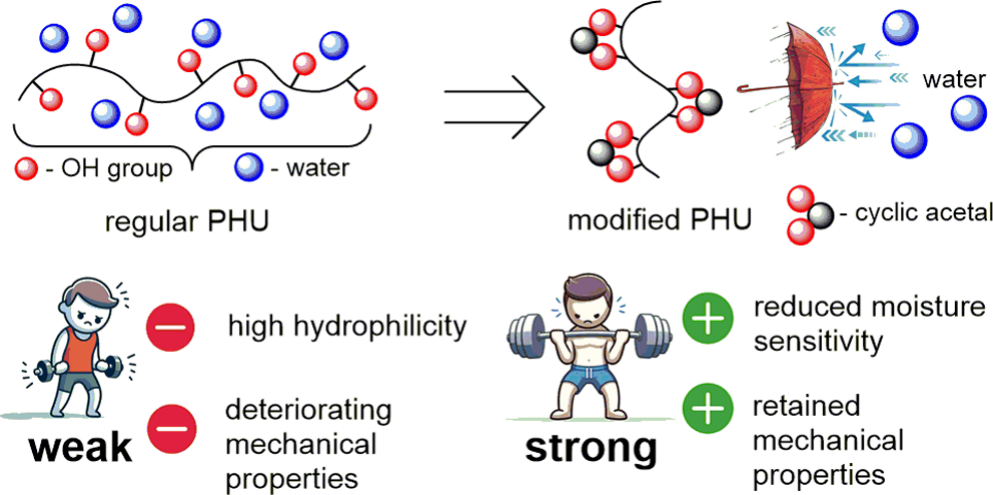

Nonisocyanate polyurethanes (NIPUs) are broadly investigated as a potential replacement for conventional polyurethanes (PUs) to eliminate the use of toxic isocyanates and reduce occupational hazards. One of the most popular approaches to NIPU synthesis is the polyaddition of cyclic bis(carbonate)s and diamines to form poly(hydroxyurethane)s (PHUs). However, such PHUs are highly hydrophilic due to the presence of two hydroxyl groups per repeat unit, and the resulting moisture absorption significantly degrades their thermomechanical performance and physical stability upon exposure to humidity, thus limiting their utility. Here, we introduce a simple and scalable approach for the modification of PHUs to increase hydrophobicity and adjust their properties. The proposed reaction between aldehydes and appropriately spaced hydroxyl groups in the polymer backbone resulted in high degrees of modification (up to 84%) and up to 3-fold reductions in water uptake at 85% RH. Furthermore, the use of aromatic aldehydes in particular enabled the retention of mechanical properties over a wide range of humidity levels, resulting in performance comparable to conventional PUs. Finally, we note that this approach is not limited to reducing moisture sensitivity alone and provides ample opportunities for imparting a broad range of novel properties to PHUs through an appropriate selection of functional aldehydes.

## Introduction

Polyurethanes (PUs) are widely used and represent a versatile family of plastics, with an annual worldwide production of 24.7 million tons in 2021.^1^ The vast array of available precursors for these materials enables a broad range of applications, including foams, coatings, adhesives, automotive parts, medical devices, sporting goods, etc.^2^ The conventional synthetic pathway to linear PUs involves the reaction of diisocyanates with diols. However, isocyanates are toxic compounds that present respiratory and dermal hazards and can cause chronic illness or even death upon overexposure.^3,4^ As a result, extensive research in academia and industry has focused on alternative synthetic pathways for the preparation of PUs that avoid the use of hazardous isocyanates.^5−10^ These so-called nonisocyanate polyurethanes (NIPUs) are considered a “greener” alternative to PUs not only due to the less hazardous monomers in use but also thanks to the availability of biobased feedstocks for their synthesis.^11−13^

**Scheme 1:**
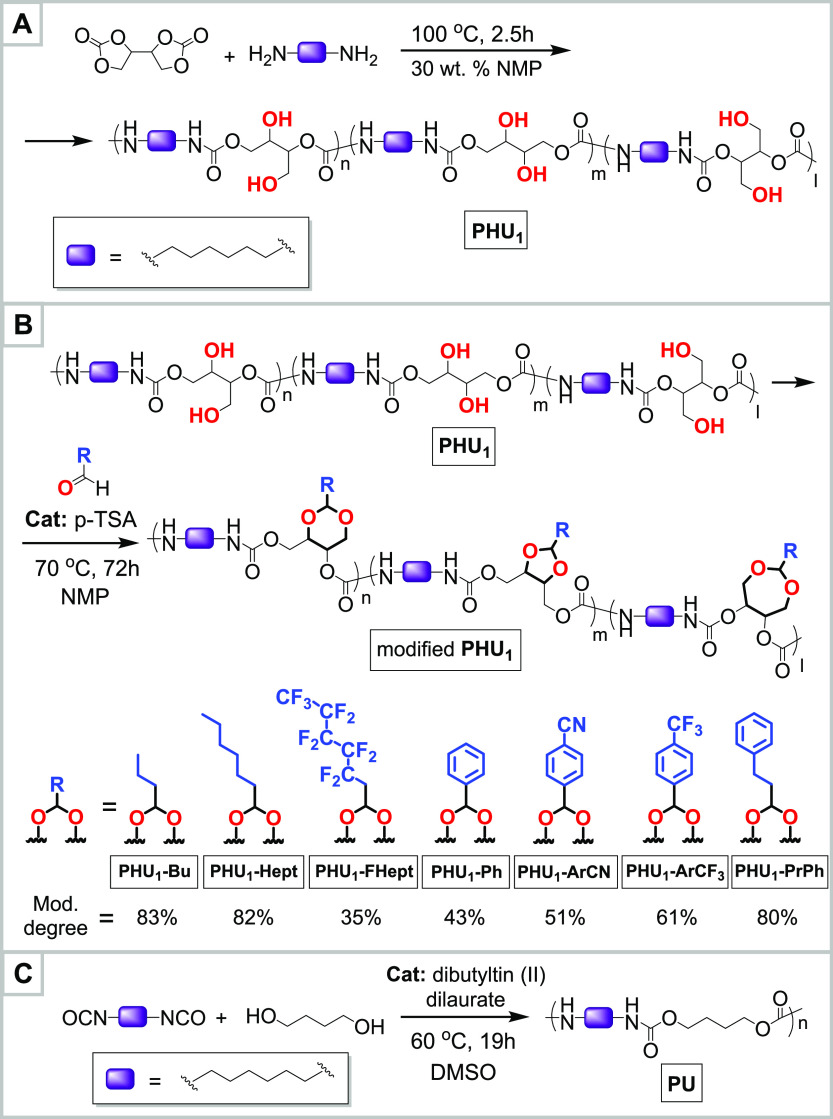
(A) **PHU**_**1**_ Synthesis from Erythritol Dicarbonate (EDC) and 1,6-Hexamethylenediamine (HMDA); (B) Modification of **PHU**_**1**_ with Selected Aliphatic and Aromatic Aldehydes; (C) Synthesis of Conventional **PU** Control from 1,6-Hexamethylene Diisocyanate (HMDI) and 1,4-Butanediol (BDO)

One of the most popular approaches for NIPU synthesis is the aminolysis of cyclic bis(carbonate)s (CCs) leading to the preparation of poly(hydroxyurethane)s (PHUs).^14,15^ This pathway is often preferred because it provides 100% atom economy and utilizes readily produced and minimally toxic CCs (Scheme 1A).

However, there are several issues inherent to PHUs that remain unresolved and limit their potential application, most notably (a) low molecular weights due to slow polymerization kinetics, low reactivity of 5-membered CCs, and the occurrence of side reactions^16,17^ and (b) significant hydrophilicity^9,18,19^ due to the presence of numerous hydroxyl groups in the polymer backbone. The substantial moisture uptake of such PHUs generally results in the deterioration of their thermomechanical properties in humid environments or upon prolonged storage and can trigger hydrolytic depolymerization during processing.

The issue of slow polymerization kinetics can be partially solved by employing reactive extrusion (ReX) to the synthesis of PHUs.^20^ Compared to conventional batch melt polycondensation^21^ and solution polymerization,^22^ ReX is able to provide the following advantages:^23^ (1) it allows for high shear rates and excellent mixing of even very viscous media in the absence of any solvents as a function of the screw geometry; (2) it ensures the highest possible reaction rates by maximizing the concentrations of monomers and initiators/catalysts (if present); (3) it reduces the reaction times required to achieve high conversion during polycondensation; (4) it facilitates heat transfer, better maintaining the reaction temperature and reducing the rate of side reactions.

To address the hydrophilicity of PHUs, in this work, we suggest a novel^24^ postsynthetic modification approach inspired by the synthesis of poly(vinyl butyral)^25^ and involving the reaction of proximate pairs of hydroxyl groups in the PHU backbone with aldehydes to form cyclic acetals (Scheme 1B). Previously, several methods for consuming these hydroxyl groups during postsynthetic modification have been reported, including esterification with acetic anhydride,^26,27^ benzoyl chloride,^27^ or chloroacetyl chloride^28^ and silylation.^27^ However, the reagents used for such modifications were either corrosive or toxic, and no studies to determine the hydrophilicity of resulting polymers were conducted. In the approach suggested in this work, the only prerequisite for polymer modification is the presence and structural availability of two adjacent hydroxyl groups in the PHU chain. This can be achieved by using tailored bis(carbonate) monomers such as erythritol dicarbonate (EDC, Scheme S1 and Figures S1 and S2) or 4-vinylcyclohexene dicarbonate (4VCHDC, Scheme S2 and Figures S3–S8) in combination with the diamine(s) of choice, thus providing the requisite pairs of proximate hydroxyls, as shown in **PHU**_**1**_ (Schemes 1A and S3) and **PHU**_**2**_ (Scheme S4).

## Results and Discussion

### PHU Synthesis and Modification

**Scheme 2:**
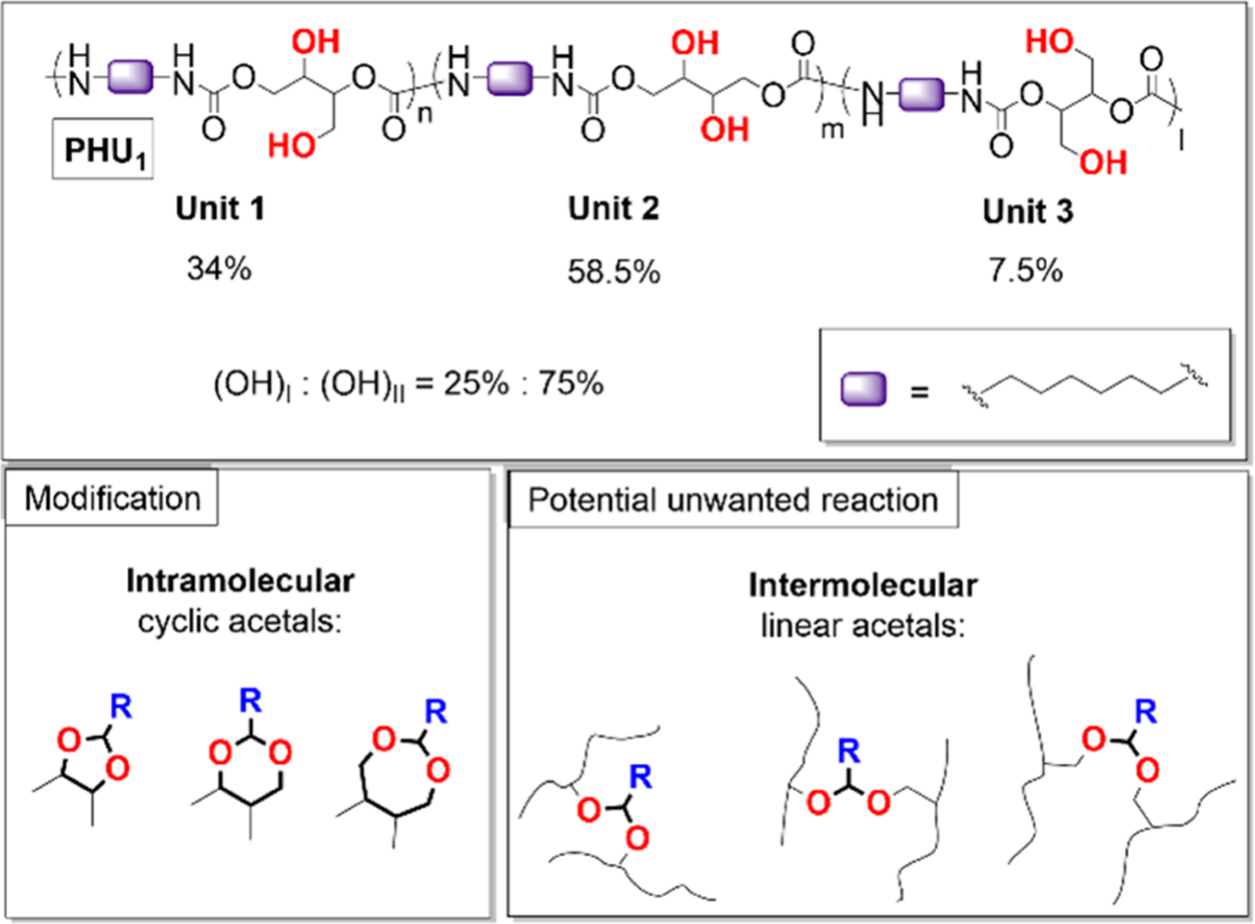
Microstructure of **PHU**_**1**_ and Structures of Intramolecular Cyclic Acetals and Intermolecular Linear Acetals That May Form during the Modification of **PHU**_**1**_

First, we performed the optimization of **PHU**_**1**_ synthesis, previously reported by Mülhaupt et al.^22^ Optimization of the reactive extrusion conditions, including reaction time and temperature, nature and amount of hydrogen bond disrupting agent,^16^ etc., (see Section SVI) enabled the preparation of linear **PHU**_**1**_ with molecular weights as high as *M*_n(SEC)_ = 15 100 g mol^–1^ and *M*_w_/*M*_n_ values in the range of 3.3–6.5. Based on a detailed analysis of all experiments in Table S1, it can be concluded that the optimal reaction conditions for the synthesis of **PHU**_**1**_ with the highest possible molecular weight and in the highest yield are as follows: 100 °C, 2.5 h, and the addition of 30 wt % of *N*-methyl-2-pyrrolidone (NMP) as a solubilizing and hydrogen bond disrupting agent (see Section SIII.1 and Figures S10–S14). The structure of **PHU**_**1**_ was confirmed by NMR following a method adapted from Cramail et al.^29^ According to NMR (Section SV.5), the ratio between first, second, and third repeat unit types (Scheme 2) was found to be equal to 34.0:58.5:7.5 mol %. This implies a total ratio of primary to secondary hydroxyl groups of (OH)_I_/(OH)_II_ = 25:75. The presence of urea moieties^20^ was also detected as a concentration of approximately 1.5 mol %.

As the next step, **PHU**_**1**_ was used as the parent polymer for the creation of a series of modified linear PHUs bearing aliphatic or aromatic substituents (Scheme 1B and Table 1). The formation of cyclic acetals was achieved through the reaction between the corresponding aldehyde and pairs of adjacent hydroxyl groups in the **PHU**_**1**_ main chain, catalyzed by *p*-toluenesulfonic acid (*p*TSA). Optimization of the reaction conditions was carried out using the modification of **PHU**_**1**_ with butyraldehyde as representative of the overall approach (see Section SVII, Table S2, and Figures S44 and S45). Varying the excess of aldehyde, the amount of *p*TSA, the temperature, and the duration of the reaction, it was possible to achieve as high degree of modification as 84% (Table 1, **PHU**_**1**_**-Bu)**. The optimal conditions were determined to be as follows: NMP as a reaction solvent, [**PHU**_**1**_] ≈ 10 wt %, 5.63 mol. equiv of aldehyde, 0.38 mol. equiv of *p*TSA, 70 °C, 72 h. The structure of the resultant **PHU**_**1**_**-Bu** was fully confirmed by NMR analysis (Section SV.1 and Figures S17–S22). Due to the complexity of the NMR spectrum, it was not possible to unambiguously attribute the signals relative to 5-, 6-, or 7-membered cyclic acetals. By taking into account the ratio between repeat unit types (58.5:34.0:7.5 mol %) determined for **PHU**_**1**_ (Scheme 2 and Section SV.5), it is expected that a similar ratio holds for 5-, 6-, and 7-membered cyclic acetals as well. However, since the reaction conversion is not quantitative and varies depending on the chosen aldehyde, the precise ratio between cyclic acetals remains somewhat uncertain. Moreover, by analogy with bulk acetalization of poly(vinyl alcohol), one can propose the formation of intermolecular acetals leading to branched structures or even cross-linked polymers (Scheme 2). It has been previously reported that such unwanted reactions can be effectively suppressed by performing the modification reaction in dilute solution rather than in bulk^25^ and through the use of reaction media and temperatures capable of suppressing hydrogen bonding^30^ – all of which applies to the current work, modification reactions having been carried out in the NMP solution at 70 °C. While the presence of intermolecular acetals cannot be proven or refuted by NMR due to the complex nature of the resultant spectra and the overlapping of the signals of interest, two observations are worth noting. First, in addition to a significant increase in solubility following modification in comparison to the parent **PHU**_**1**_ (Section SV.3 and Table S2), the lack of a gel fraction in the modified PHUs rules out significant cross-linking.^25^ The decrease in the breadth of the molecular weight distribution (*M*_w_/*M*_n_ = 8.05 (**PHU**_**1**_), 6.77 (**PHU**_**1**_**-Hept**), 5.58 (**PHU**_**1**_**-ArCN**)) following modification (Figure S81) rules out significant branching.^30^ Taken together, these observations are consistent with intramolecular cyclic acetal formation as the predominant modification reaction.

**Table 1:** Properties of Modified PHUs vs Controls **PHU**_**1**_ and **PU**

	precipitated powders					melt processed bars									films
									water uptake (wt %)^g^			*G*′ (MPa)^h^			
sample	mod. (%)^a^	*T*_g_ (*T*_m_) (°C)^b^	*T*_g_ (*T*_m_) (°C)^c^	*X*_c_ (%)^d^	*T*_onset_ (°C)^e^	*T*_α_ (°C)^f^	*E*′ (MPa)^f^	*X*_c_ (%)^d^	45% RH	65% RH	85% RH	45% RH	65% RH	85% RH	water contact angle (deg)^i^
**PHU**_**1**_		50 (97, 130)	58 (104, 134)	14.8	185	81	1690	10.3	2.0	3.7	7.5	1600	360	54	91.7 ± 0.8
**PHU**_**1**_**-Bu**	84	41	44 (108)	0.3	185	56	1660	0	1.1	2.1	3.6	210	53	2.7	106.7 ± 1.1
**PHU**_**1**_**-Hept**	84	33	36 (111)	0.7	215	45	1270	0.2	1.1	1.9	3.0	89	24	2.5	109.3 ± 0.4
**PHU**_**1**_**-FHept**	35^j^	34	42 (111)	3.6	155	k	k	k	k	k	k	k	k	k	110.0 ± 0.3
**PHU**_**1**_**-Ph**	43	43 (94)	50 (100)	5.6	205	86	1530	1.9	0.7	1.7	3.7	600	210	140	96.4 ± 0.6
**PHU**_**1**_**-ArCN**	51	52 (115)	55 (119)	6.3	175	88	2360	3.4	1.0	1.9	4.7	770	450	56	95.6 ± 0.6
**PHU**_**1**_**-ArCF**_**3**_	61	57	59	0	195	71	1610	0	0.8	1.5	3.2	530	330	200	97.3 ± 0.4
**PHU**_**1**_**-PrPh**	80	42	46	0	225	51	1310	0	0.2	1.1	2.4	420	330	74	89.8 ± 0.7
**PU**^l^		(187)	45 (188)	56.0	200	42	2200	35.2	0.4	0.4	1.1	460	400	300	95.1 ± 3.0

a By ^1^H NMR (see Supporting Information).

b By DSC in N_2_ at a heating rate of 5 °C/min; *T*_m_ was taken as peak maximum.

c By DSC in N_2_ at a heating rate of 10 °C/min; *T*_m_ was taken as peak maximum.

d Degree of crystallinity determined by DSC in N_2_ at a heating rate of 10 °C/min (see Supporting Information).

e By TGA in air at a heating rate of 5 °C/min.

f Storage modulus (*E*′) and *T*_α_ (taken as peak maximum from the loss modulus (*E*″) curve) measured by DMTA at a heating rate of 5 °C/min.

g Determined after 11 days of conditioning at selected humidity levels and 22 °C.

h Storage modulus (*G*′) at 0.1 Hz measured via torsional rheometry at 25 °C and indicated humidity level after conditioning at selected humidity levels and 22 °C for 3 days.

i Water contact angle measured on films at 22 °C.

j By ^19^F NMR with external standard (C_6_F_6_).

k Not determined due to low extent of modification.

l For comparison.

Building on this work, various aldehydes (both aliphatic and aromatic) were used to modify the parent compound **PHU**_**1**_ (Scheme 1B and Section SV.2). The degree of modification was found to be dependent on the nature of the aldehyde and was determined using NMR (^1^H or ^19^F; Section SV.3). The utilization of compounds containing nonaromatic aldehyde groups (Table 1, **PHU**_**1**_**-Bu**, **PHU**_**1**_**-Hept**, and **PHU**_**1**_**-PrPh**) resulted in high degrees of modification ranging from 80 to 84%. Fluorinated 3,3,4,4,5,5,6,6,7,7,7-undecafluoroheptanal (F-heptaldehyde), in contrast, gave only 35% modification (Table 1, **PHU**_**1**_**-FHept**) probably due to unwanted oligomerization of the F-heptaldehyde. Compounds containing aromatic aldehyde groups demonstrated a lower reactivity in comparison to that of their aliphatic counterparts (Table 1, **PHU**_**1**_**-Ph**). While this can be explained by a combination of steric and electronic effects, the latter seems to predominate, as deduced from the comparison between **PHU**_**1**_**-Ph** (43% degree of modification) and **PHU**_**1**_**-PrPh** (80% degree of modification). In aromatic aldehydes, the lone pair of electrons on the carbonyl oxygen can be delocalized by the benzene ring through resonance, thus making the electron density on the oxygen less available for nucleophilic attack and decreasing the success of modification as in the case of **PHU**_**1**_**-Ph**. The introduction of the aliphatic bridge between the aldehyde function and the benzene ring as in 3-phenylpropionaldehyde suppresses this resonance and increases the degree of modification. However, the aromatic aldehydes can be activated through the introduction of electron-withdrawing substituents in the *para* position of the benzene ring, thus yielding increases in the extent of modification from 43 to 51 and 61% for **PHU**_**1**_**-Ph**, **PHU**_**1**_**-ArCF**_**3**_, and **PHU**_**1**_**-ArCN**, respectively (Table 1).

To further confirm these trends, another parent PHU (**PHU**_**2**_) having two adjacent hydroxyl groups was modified with 3-phenylpropionaldehyde (Scheme S13 and Section SV.1.8) under optimal reaction conditions, yielding an extent of modification as high as 50%. This lower value was anticipated due to the larger spacing between the hydroxyl functions (Scheme S13), given that the formation of cyclic acetals with more than 7 carbon atoms is believed to be less favorable. The large distances present between the hydroxyl groups in some structural isomers make it impossible to form cyclic acetals and lead to further decreases in the extent of modification. Nevertheless, this example one more time demonstrates the applicability of the method given the existence of appropriate structures in the parent PHU. Finally, for comparison purposes, a conventional polyurethane **PU** with a structure analogous to that of **PHU**_**1**_ was prepared via the reaction of 1,6-hexamethylene diisocyanate and 1,4-butanediol (Scheme 1C).

### PHU Properties in a Dry State

Following their preparation, the thermal and mechanical properties of the modified PHUs were assessed using DSC (Figures S46–S65), DMTA (Figure S67), and TGA methods. Modification with compounds containing aliphatic aldehyde groups led to small decreases in the glass transition temperature (Table 1, **PHU**_**1**_-**Bu**, **PHU**_**1**_-Hept, and **PHU**_**1**_**-PrPh**), with the magnitude of the decrease observed to be proportional to the length of the side chain (Table 1, **PHU**_**1**_**-Bu** and **PHU**_**1**_**-Hept**) at a comparable degree of modification. In contrast, the use of compounds containing aromatic aldehyde groups resulted in similar (**PHU**_**1**_**-Ph**, **PHU**_**1**_**-ArCN**) or higher *T*_g_ values (in the case of **PHU**_**1**_**-ArCF**_**3**_) vs the parent **PHU**_**1**_.

These materials were additionally observed to be semicrystalline as well (Table 1), with **PHU**_**1**_**-FHept** (Figure S57), **PHU**_**1**_**-Ph** (Figure S59), and **PHU**_**1**_**-ArCN** (Figure S61) displaying DSC endotherms at ∼100 °C attributed to the melting of the crystalline phase.^31^ The highest degree of crystallinity (∼56%) was observed in the control PU (Figure S51), in full agreement with published data.^31,32^ A modest degree of crystallinity (∼15%) was observed in parent **PHU**_**1**_ (Figure S48), while PHUs modified with compounds containing aromatic aldehyde groups showed even lower levels of crystallinity (∼6%). Finally, PHUs modified with compounds bearing aliphatic aldehyde groups displayed the lowest levels of crystallinity (∼0.5%). While all attempts to crystallize these materials in subsequent DSC cycles failed, dissolution of the amorphous (melted) PHU in THF and repeated precipitation in Et_2_O resulted in the regeneration of semicrystalline material. The cooling of the modified PHUs in the compression mold under vacuum induced crystallinity as well, albeit to a lesser extent than in the case of solvent-precipitated material (Table 1). The observed crystallinity was further confirmed (qualitatively) by WAXD analysis (Figures S67–S70).

**Figure 1:**
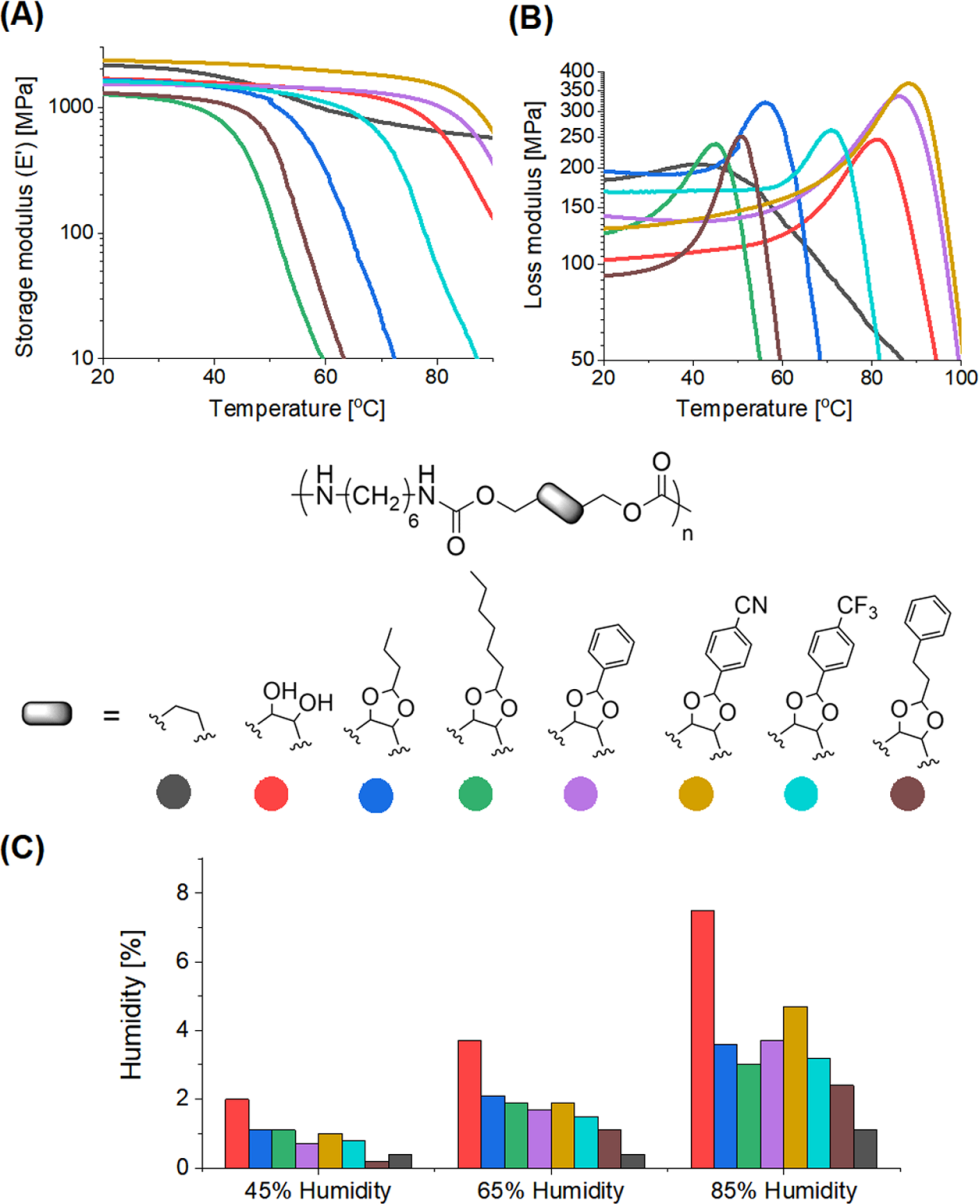
Temperature dependence of storage (*E*′) (A) and loss moduli (*E*″) (B) for modified PHUs, **PHU**_**1**_, and **PU** samples measured by DMTA; room temperature water uptake of modified PHUs, **PHU**_**1**_, and **PU** at 45, 65, and 85% RH (C).

DMTA analyses were performed on polymer bars in tension mode (Figures 1A,B and S66). The observed *T*_g_ (*T*_α_ more exactly) values for **PHU**_**1**_ and all modified PHUs were slightly higher than those determined by DSC with the same heating rate (Table 1). Nevertheless, the tendency in PHUs *T*_g_ was found to be in good agreement with DSC. The storage modulus (E’) of the modified PHUs is presented in Figure 1A and Table 1. The glassy plateau of the majority of the modified PHUs is higher than that of the parent **PHU**_**1**_ but generally lower than that of the conventional **PU**. Only the PHU modified with 4-formylbenzonitrile displayed a glassy plateau comparable to that of **PU** (Table 1, **PHU**_**1**_**-ArCN**). Nevertheless, these results confirm that the modification method presented here can provide similar or greater levels of thermomechanical performance vs the parent **PHU**_**1**_.

The tensile properties of selected PHUs were studied as well (Section SXIV, Table S6, and Figure S82). While the tensile strength decreased from 20.7 ± 1.1 MPa for parent **PHU**_**1**_ to 10.2 ± 1.8 for **PHU**_**1**_**-Bu** and 6.7 ± 0.7 for **PHU**_**1**_**-ArCF**_**3**_, the elongation at break was found to be less dependent on the PHU structure (Figure S82), with similar results for **PHU**_**1**_ (1.77 ± 0.26%) and **PHU**_**1**_**-Bu** (1.79 ± 0.19%) and slightly lower values for **PHU**_**1**_**-ArCF**_**3**_ (0.80 ± 0.09%). Such shifts in properties are most readily explained by a reduction in the level of intermolecular hydrogen bonding since the majority of pendant hydroxyl groups are involved in the formation of cyclic acetal groups, and are entirely consistent with early reports of decreases in tensile strength with increasing degree of acetalization in poly(vinyl alcohol) as well.^33^

### PHU Properties at Different Humidities

The next step in this work consisted of the investigation of the PHU properties as a function of the relative humidity (RH). First, the room temperature water uptake of modified PHUs was studied at three humidity levels (45, 65, and 85% RH) and compared to that of parent **PHU**_**1**_ and conventional **PU** (Table 1 and Figures 1C and S71 and S72). The consumption of the free hydroxyl groups in the modified PHUs resulted in significant reductions in hydrophilicity by a factor of 2–3 times as compared to neat **PHU**_**1**_. The most pronounced effect was observed for **PHU**_**1**_**-PrPh**, which displayed only 2.4% of water uptake after 11 days of conditioning at 85% RH vs 7.5% for the parent **PHU**_**1**_. The smallest improvement was observed in the case of **PHU**_**1**_**-ArCN**, probably due to the presence of polar CN groups. To ensure that the sorption of water does not lead to hydrolysis of the modified PHUs, a comparative study of dry samples and those conditioned for 4 weeks at 85% RH was performed via GPC and NMR (Section SXIII and Figures S76–S79). Figure S80 undoubtedly demonstrates that there is no change in *M*_w_ as a consequence of exposure to moisture. Consistent with this observation, NMR spectra (Figures S76–S78) show that the degree of modification remains unchanged after prolonged conditioning as well.

**Figure 2:**
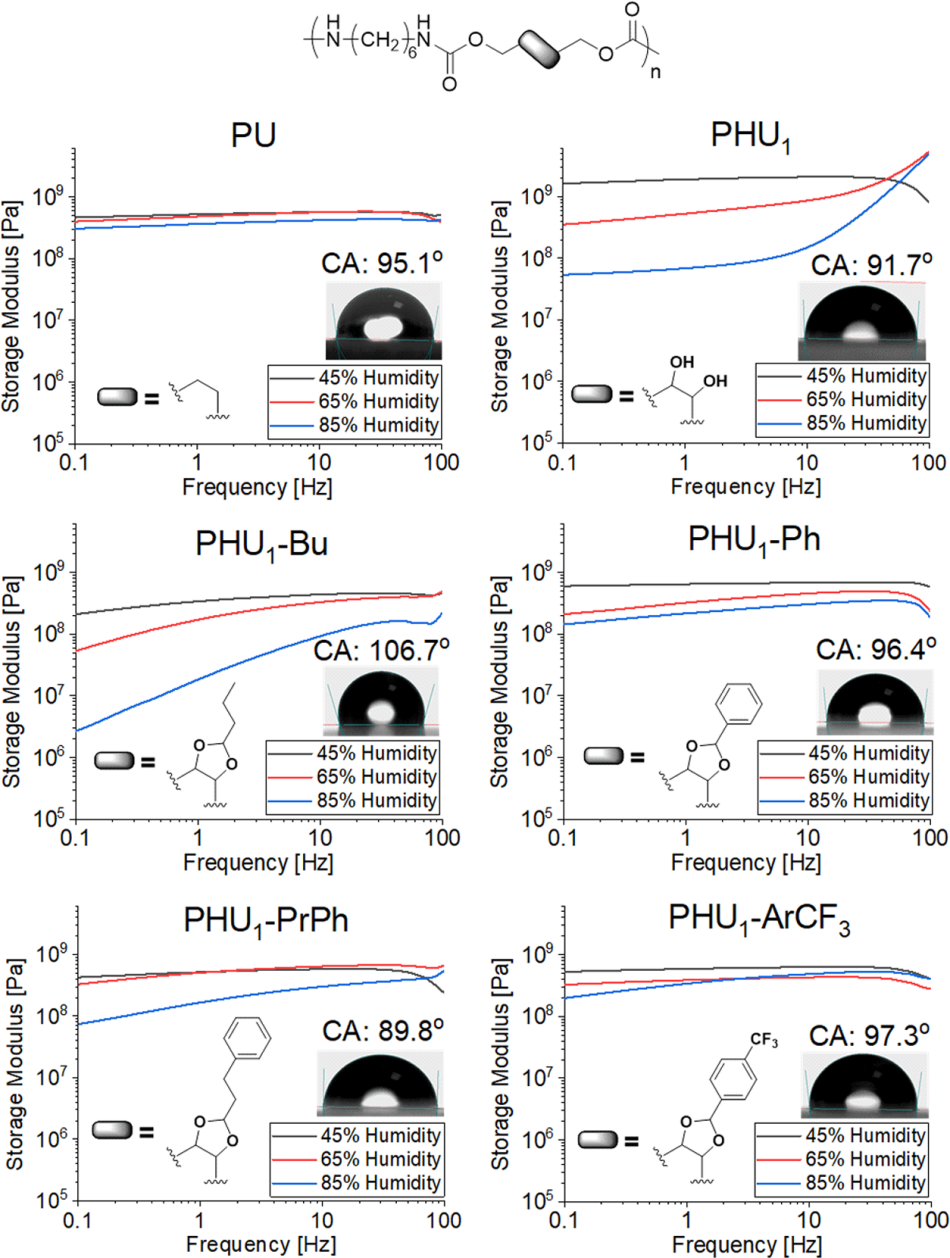
Frequency dependence of storage moduli (*G*′) for modified PHUs, **PHU**_**1**_, and **PU** controls, measured after conditioning at different humidity levels and wettability (water contact angles, CAs) of respective polymer films.

Similar trends were observed in an investigation of water contact angles (Table 1 and Figure 2). The modification of **PHU**_**1**_ resulted in a decrease in the wettability of the polymer surface and an increase in contact angles from 91.7° to as high as 109.3°. The most pronounced improvement was observed for PHUs modified with aldehydes containing (fluorinated) linear hydrocarbon segments (Table 1, **PHU**_**1**_**-Bu**, **PHU**_**1**_**-Hept**, and **PHU**_**1**_**-FHept**), where an increase in the length of the alkyl tail resulted in an increase in CA (Table 1, **PHU**_**1**_**-Bu** and **PHU**_**1**_**-Hept)**.

Such modifications resulted in reduced wettability not only in comparison to the parent hydrophilic **PHU**_**1**_ but also vs conventional **PU**. The modification of **PHU**_**1**_ with compounds containing aromatic substituents also increased the measured water contact angles but to a lesser extent (Table 1, **PHU**_**1**_**-Ph**, **PHU**_**1**_**-ArCF**_**3**_, and **PHU**_**1**_**-ArCN**), with the most pronounced effect for the aromatic substituent containing a CF_3_ group. An exception was observed for **PHU**_**1**_**-PrPh**, which, despite a high extent of modification, gave a CA quite similar to that of the parent **PHU**_**1**_ and below that of both **PHU**_**1**_**-Bu** and **PHU**_**1**_**-Ph**. As this result would not be expected based on structural arguments alone, one possible explanation is that this material experiences strong local interactions with liquid water, enabling reorganization of the polymer surface (during the formation of the film) to display only the most polar functional groups. This is consistent with the much larger drop in *G*′ observed in this system vs **PHU**_**1**_**-Ph** as RH is increased and may be further aided by the limited ability of the propylphenyl substituents to self-associate and pack space efficiently in contrast to linear alkyl substituents like those present in **PHU**_**1**_**-Bu** for instance. While the in-depth studies necessary to fully understand this phenomenon are beyond the scope of the current report, it is nevertheless noteworthy that water-induced surface reorganization has long been reported in the literature for some of the conventional polyurethanes.^34−37^

The mechanical properties vs humidity for modified PHUs were studied via torsional rheometry on polymer bars (Figures 2 and S73–S75). The samples were conditioned for 3 days at the selected humidity level and were then characterized in torsion mode at room temperature while maintaining the same humidity levels inside the rheometer chamber. The storage modulus (*G*′) values were found to display only modest decreases with increasing humidity for conventional **PU**. In contrast, much larger decreases were observed in the case of **PHU**_**1**_ (Figure 2), with *G*′ reduced by nearly 2 orders of magnitude when moving from 45 to 85% RH. Data taken after 14 days of conditioning were similar (Figure S76), implying that 3 days was sufficient to realize equilibrium moisture uptake in these specimens. This large difference in the moisture sensitivity of the elastic response is explained by the presence of large concentrations of hydrophilic OH groups in **PHU**_**1**_ leading to significant water uptake (Figure 1C) and subsequent plasticization of the polymer by the absorbed water. The rheometer data for the modified PHUs (Figure 2) showed opposing trends as a function of the aldehyde type. While the use of aliphatic aldehydes resulted in even larger decreases in *G*′ with increasing humidity as compared with the parent **PHU**_**1**_ (Figure 2, **PHU**_**1**_**-Bu**), the use of aldehydes containing aromatic rings led to substantial reductions in the sensitivity of the modulus to humidity (Figure 2, **PHU**_**1**_**-Ph**, **PHU**_**1**_**-ArCF**_**3**_, and **PHU**_**1**_**-PrPh**).

For example, the storage moduli of **PHU**_**1**_**-PrPh** at 45 and 65% RH show only modest differences, with a large decrease not observed until 85% RH. The behavior of **PHU**_**1**_**-Bu** and **PHU**_**1**_**-Hept** is most obviously explained by the low *T*_g_ (41 and 33 °C, respectively) and lack of crystallinity apparently induced by modification with compounds containing aromatic aldehydes. In such materials, the absorption of even small amounts of water will plasticize the entire mass of the polymer, reducing the *T*_g_ to values below RT and leading to much more significant softening than in the case of materials with either higher initial *T*_g_ values or some level of crystallinity (Table 1, **PHU**_**1**_**-Ph**, **PHU**_**1**_**-ArCN**, and **PHU**_**1**_**-ArCF**_**3**_). In contrast, the use of aromatic aldehydes provides modified PHUs with the most consistent behavior vs humidity (Figure 2, **PHU**_**1**_**-Ph**, **PHU**_**1**_**-ArCN**, and **PHU**_**1**_**-ArCF**_**3**_). The decrease in the storage modulus for **PHU**_**1**_**-ArCF**_**3**_ was found to be only 2.5-fold between 45 and 85% RH and is comparable to the 1.5-fold decrease for conventional **PU**. Surprisingly, **PHU**_**1**_**-Ph**, which has only 43% of modification, was also able to retain its mechanical properties after exposure to 85% RH, displaying a 4-fold decrease in storage modulus (slightly less than that for **PHU**_**1**_**-PrPh**). This could be explained by the presence of crystallinity in **PHU**_**1**_**-Ph** compared to the fully amorphous nature of **PHU**_**1**_**-PrPh** and by the relative lack of mobility of the phenyl substituents in the former case as compared to the propylphenyl substituents in the latter case.

Finally, it was observed that the storage moduli (*G*′) of **PHU**_**1**_ and to a lesser extent **PHU**_**1**_**-Bu** and **PHU**_**1**_**-PrPh** show a greater frequency dependence at elevated RH levels as compared to the other materials studied (Figure 2). This behavior would seem to be best explained by a change in *T*_α_ due to plasticization with absorbed water, as has been estimated using the Fox equation^38^ (Section SXII and Table S5). These calculations reveal that at the highest RH level (85%) three of the five PHUs studied (**PHU**_**1**_, **PHU**_**1**_**-Bu**, and **PHU**_**1**_**-PrPh**) are expected to give α transition temperatures of ∼40 °C vs ∼55 °C for **PHU**_**1**_**-ArCF**_**3**_ and ∼65 °C for **PHU**_**1**_**-Ph**. While such results are necessarily approximate, they indicate that the hydration of **PHU**_**1**_, **PHU**_**1**_**-Bu**, and **PHU**_**1**_**-PrPh** results in a decrease in the main relaxation temperature to the point where it is close enough to the measurement temperature that the modulus begins to exhibit a significant frequency dependence (as expected near the main relaxation). In contrast, the **PU** control does not exhibit such behavior in spite of its similarly low *T*_α_ of ∼40 °C due to its much higher crystalline fraction (∼35%). Here, the high-stiffness crystalline fraction dominates the mechanical response of the **PU** and provides a higher, less frequency-dependent modulus vs PHUs with the same main relaxation temperature but much lower levels of crystallinity (∼0–10%).

## Conclusions

In this work, we have shown that specifically tailored PHUs with proximate hydroxyl groups in the backbone can be readily modified by a simple reaction with aldehydes. The suggested approach allows for PHU modification that significantly improves hydrophobicity and reduces the impact of humidity on the mechanical response. Moreover, keeping in mind the ready availability of a broad range of available functional aldehydes, this method provides an easy means of imparting novel/application-specific properties to PHUs, greatly increasing their potential utility.

## Supporting Information Available

Additional experimental details including materials and methods; synthesis of cyclic carbonate monomers; **PHU_1_** and **PHU_2_** synthesis and modification; PU synthesis; determination of extent of PHU modification using NMR; determination of crystallinity degree in polymers; discussion and analysis including optimization of PHU synthesis and modification; DSC plots; DMTA plots; representative WAXD plots; water absorption plots; storage moduli (*G*′) plots at different humidity levels; GPC plots and tensile plots (DOCX)

## References

[ref1] Statista. Market Value of Polyurethane Worldwide from 2015 to 2021, with a Forecast for 2022 to 2029. https://www.statista.com/statistics/720341/global-polyurethane-market-size-forecast/ (accessed March 27, 2023).

[ref2] Das A.; Mahanwar P. A Brief Discussion on Advances in Polyurethane Applications. Adv. Ind. Eng. Polym. Res. 2020, 3 (3), 93–101. 10.1016/j.aiepr.2020.07.002.

[ref3] OSHWiki. Isocyanates. https://oshwiki.osha.europa.eu/en/themes/isocyanates (accessed March 27, 2023).

[ref4] Kimber I.; Dearman R. J.; Basketter D. A. Diisocyanates, Occupational Asthma and IgE Antibody: Implications for Hazard Characterization. J. Appl. Toxicol. 2014, 34 (10), 1073–1077. 10.1002/jat.3041. 25059672

[ref5] Maisonneuve L.; Lamarzelle O.; Rix E.; Grau E.; Cramail H. Isocyanate-Free Routes to Polyurethanes and Poly(Hydroxy Urethane)S. Chem. Rev. 2015, 115 (22), 12407–12439. 10.1021/acs.chemrev.5b00355. 26516788

[ref6] Grignard B.; Gennen S.; Jérôme C.; Kleij A. W.; Detrembleur C. Advances in the Use of CO 2 as a Renewable Feedstock for the Synthesis of Polymers. Chem. Soc. Rev. 2019, 48 (16), 4466–4514. 10.1039/C9CS00047J. 31276137

[ref7] Rokicki G.; Parzuchowski P. G.; Mazurek M. Non-Isocyanate Polyurethanes: Synthesis, Properties, and Applications. Polym. Adv. Technol. 2015, 26 (7), 707–761. 10.1002/pat.3522.

[ref8] Gomez-Lopez A.; Elizalde F.; Calvo I.; Sardon H. Trends in Non-Isocyanate Polyurethane (NIPU) Development. Chem. Commun. 2021, 57 (92), 12254–12265. 10.1039/D1CC05009E. 34709246

[ref9] Gomez-Lopez A.; Panchireddy S.; Grignard B.; Calvo I.; Jerome C.; Detrembleur C.; Sardon H. Poly(Hydroxyurethane) Adhesives and Coatings: State-of-the-Art and Future Directions. ACS Sustainable Chem. Eng. 2021, 9 (29), 9541–9562. 10.1021/acssuschemeng.1c02558. 35692866 PMC9173693

[ref10] Khatoon H.; Iqbal S.; Irfan M.; Darda A.; Rawat N. K. A Review on the Production, Properties and Applications of Non-Isocyanate Polyurethane: A Greener Perspective. Prog. Org. Coat. 2021, 154, 106124 10.1016/j.porgcoat.2020.106124.

[ref11] Carré C.; Ecochard Y.; Caillol S.; Avérous L. From the Synthesis of Biobased Cyclic Carbonate to Polyhydroxyurethanes: A Promising Route towards Renewable Non-Isocyanate Polyurethanes. ChemSusChem 2019, 12 (15), 3410–3430. 10.1002/cssc.201900737. 31099968

[ref12] Ghasemlou M.; Daver F.; Ivanova E. P.; Adhikari B. Bio-Based Routes to Synthesize Cyclic Carbonates and Polyamines Precursors of Non-Isocyanate Polyurethanes: A Review. Eur. Polym. J. 2019, 118, 668–684. 10.1016/j.eurpolymj.2019.06.032.

[ref13] Pelckmans M.; Renders T.; Van de Vyver S.; Sels B. F. Bio-Based Amines through Sustainable Heterogeneous Catalysis. Green Chem. 2017, 19 (22), 5303–5331. 10.1039/C7GC02299A.

[ref14] Sardon H.; Pascual A.; Mecerreyes D.; Taton D.; Cramail H.; Hedrick J. L. Synthesis of Polyurethanes Using Organocatalysis: A Perspective. Macromolecules 2015, 48 (10), 3153–3165. 10.1021/acs.macromol.5b00384.

[ref15] Cornille A.; Auvergne R.; Figovsky O.; Boutevin B.; Caillol S. A Perspective Approach to Sustainable Routes for Non-Isocyanate Polyurethanes. Eur. Polym. J. 2017, 87, 535–552. 10.1016/j.eurpolymj.2016.11.027.

[ref16] Blain M.; Cornille A.; Boutevin B.; Auvergne R.; Benazet D.; Andrioletti B.; Caillol S. Hydrogen Bonds Prevent Obtaining High Molar Mass PHUs. J. Appl. Polym. Sci. 2017, 134 (45), 44958 10.1002/app.44958.

[ref17] Besse V.; Camara F.; Méchin F.; Fleury E.; Caillol S.; Pascault J.; Boutevin B. How to Explain Low Molar Masses in PolyHydroxyUrethanes (PHUs). Eur. Polym. J. 2015, 71, 1–11. 10.1016/j.eurpolymj.2015.07.020.

[ref18] Dong T.; Dheressa E.; Wiatrowski M.; Pereira A. P.; Zeller A.; Laurens L. M. L.; Pienkos P. T. Assessment of Plant and Microalgal Oil-Derived Nonisocyanate Polyurethane Products for Potential Commercialization. ACS Sustain.able Chem. Eng. 2021, 9 (38), 12858–12869. 10.1021/acssuschemeng.1c03653.

[ref19] Tomita H.; Sanda F.; Endo T. Polyaddition Behavior of Bis(Five- and Six-Membered Cyclic Carbonate)s with Diamine. J. Polym. Sci., Part A: Polym. Chem. 2001, 39 (6), 860–867. 10.1002/1099-0518(20010315)39:6<860::AID-POLA1059>3.0.CO;2-2.

[ref20] Magliozzi F.; Chollet G.; Grau E.; Cramail H. Benefit of the Reactive Extrusion in the Course of Polyhydroxyurethanes Synthesis by Aminolysis of Cyclic Carbonates. ACS Sustainable Chem. Eng. 2019, 7, 17282–17292. 10.1021/acssuschemeng.9b04098.

[ref21] Wołosz D.; Fage A. M.; Parzuchowski P. G.; Świderska A.; Brüll R. Reactive Extrusion Synthesis of Biobased Isocyanate-Free Hydrophobically Modified Ethoxylated Urethanes with Pendant Hydrophobic Groups. ACS Sustainable Chem. Eng. 2022, 10 (35), 11627–11640. 10.1021/acssuschemeng.2c03535. 36092287 PMC9450225

[ref22] Schmidt S.; Gatti F. J.; Luitz M.; Ritter B. S.; Bruchmann B.; Mülhaupt R. Erythritol Dicarbonate as Intermediate for Solvent- and Isocyanate-Free Tailoring of Bio-Based Polyhydroxyurethane Thermoplastics and Thermoplastic Elastomers. Macromolecules 2017, 50 (6), 2296–2303. 10.1021/acs.macromol.6b02787.

[ref23] Fink J. K. Reactive Extrusion. In Reactive Polymers: Fundamentals and Applications; Elsevier, 2018; pp 449–496.

[ref24] Shaplov A. S.; Schmidt D.; Zubkevich S. Method for Modification of Poly(Hydroxyurethanes) (PHUs). Luxembourg Patent LU503467, 2023.

[ref25] Fernández M. D.; Fernández M. J.; Hoces P. Synthesis of Poly (Vinyl Butyral)s in Homogeneous Phase and Their Thermal Properties. J. Appl. Polym. Sci. 2006, 102 (5), 5007–5017. 10.1002/app.25004.

[ref26] Beniah G.; Heath W. H.; Torkelson J. M. Functionalization of Hydroxyl Groups in Segmented Polyhydroxyurethane Eliminates Nanophase Separation. J. Polym. Sci., Part A: Polym. Chem. 2017, 55 (20), 3347–3351. 10.1002/pola.28722.

[ref27] Ochiai B.; Inoue S.; Endo T. One-Pot Non-Isocyanate Synthesis of Polyurethanes from Bisepoxide, Carbon Dioxide, and Diamine. J. Polym. Sci., Part A: Polym. Chem. 2005, 43 (24), 6613–6618. 10.1002/pola.21103.

[ref28] Matsukizono H.; Endo T. Synthesis of Polyhydroxyurethanes from Di(Trimethylolpropane) and Their Application to Quaternary Ammonium Chloride-Functionalized Films. RSC Adv. 2015, 5 (87), 71360–71369. 10.1039/C5RA09885H.

[ref29] Salvado V.; Dolatkhani M.; Grau É.; Vidil T.; Cramail H. Sequence-Controlled Polyhydroxyurethanes with Tunable Regioregularity Obtained from Sugar-Based Vicinal Bis-Cyclic Carbonates. Macromolecules 2022, 55 (16), 7249–7264. 10.1021/acs.macromol.2c01112.

[ref30] Rumyantsev M.; Rumyantsev S.; Kazantsev O. A.; Kamorina S. I.; Korablev I. A.; Kalagaev I. Y. Managing of Hydrogen Bonding in Aqueous Poly(Vinyl Alcohol) Solutions: New Perspectives towards Preparation of More Homogeneous Poly(Vinyl Butyral). J. Polym. Res. 2020, 27 (3), 52 10.1007/s10965-020-2031-y.

[ref31] Kajiyama T.; MacKnight W. J. Thermal Properties of Polyurethanes. Enthalpies and Entropies of Fusion. Polym. J. 1970, 1 (5), 548–554. 10.1295/polymj.1.548.

[ref32] Marín R.; Muñoz-Guerra S. Linear Polyurethanes Made from Threitol: Acetalized and Hydroxylated Polymers. J. Polym. Sci., Part A: Polym. Chem. 2008, 46 (24), 7996–8012. 10.1002/pola.23099.

[ref33] Fitzhugh A. F.; Crozier R. N. Relation of Composition of Polyvinyl Acetals to Their Physical Properties. I. Acetals of Saturated Aliphatic Aldehydes. J. Polym. Sci. 1952, 8 (2), 225–241. 10.1002/pol.1952.120080208.

[ref34] Tingey K. G.; Andrade J. D. Probing Surface Microheterogeneity of Poly(Ether Urethanes) in an Aqueous Environment. Langmuir 1991, 7 (11), 2471–2478. 10.1021/la00059a013.

[ref35] Xu L.-C.; Runt J.; Siedlecki C. A. Dynamics of Hydrated Polyurethane Biomaterials: Surface Microphase Restructuring, Protein Activity and Platelet Adhesion. Acta Biomater. 2010, 6 (6), 1938–1947. 10.1016/j.actbio.2009.11.031. 19948255

[ref36] Takahara A.; Takahashi K.; Kajiyama T. Effect of Polyurethane Surface Chemistry on Its Lipid Sorption Behavior. J. Biomater. Sci., Polym. Ed. 1994, 5 (3), 183–196. 10.1163/156856293X00285. 8155607

[ref37] Agnihotri A.; Garrett J. T.; Runt J.; Siedlecki C. A. Atomic Force Microscopy Visualization of Poly(Urethane Urea) Microphase Rearrangements under Aqueous Environment. J. Biomater. Sci., Polym. Ed. 2006, 17 (1–2), 227–238. 10.1163/156856206774879036. 16411611

[ref38] Fox T. G. Influence of Diluent and of Copolymer Composition on the Glass Temperature of a Polymer System. Bull. Am. Phys. Soc. 1956, 1, 123.

