# Supplementary material for: Unique Method for Facile Postsynthetic Modification of Nonisocyanate Polyurethanes
Source: Macromolecules. 2024 Feb 29;57(5):2385–93. doi: 10.1021/acs.macromol.3c02232 (PMC10938877; doi:10.1021/acs.macromol.3c02232)
Supplement: Supplementary file 1 — ma3c02232_si_001.docx [file ma3c02232_si_001.docx]

**Supporting information for**

Unique method for facile post-synthetic modification of non-isocyanate polyurethanes

Sergei V. Zubkevich^†^, Maksim Makarov^†^, Reiner Dieden^†^, Laura Puchot^†^, Vincent Berthé^†^, Stephan Westermann^†^, Alexander S. Shaplov*^,†^ and Daniel F. Schmidt*^,†^

^†^ Luxembourg Institute of Science and Technology (LIST), 5, Avenue des Hauts-Fourneaux, L-4362 Esch-sur-Alzette, Luxembourg. E-mails: [alexander.shaplov@list.lu](mailto:alexander.shaplov@list.lu); [daniel.schmidt@list.lu](mailto:daniel.schmidt@list.lu)

**Contents**

[**A.**	**Experimental part**	2](#_Toc154010767)

[I.	Materials and methods	2](#_Toc154010768)

[I.1. Materials	2](#_Toc154010769)

[I.2. Methods	3](#_Toc154010770)

[II.	Monomer synthesis.	5](#_Toc154010771)

[II.1. Erythritol dicarbonate (EDC)	5](#_Toc154010772)

[II.2. 4-Vinylcyclohexene dicarbonate (4VCHBC)	7](#_Toc154010773)

[III.	Polyhydroxyurethane (PHU) synthesis.	11](#_Toc154010774)

[III.1. Synthesis of PHU based on EDC and HMDA (**PHU_1_**).	11](#_Toc154010775)

[III.2. Synthesis of PHU based on 4VCHBC and HMDA (**PHU_2_**).	15](#_Toc154010776)

[IV.	Polyurethane (PU) synthesis.	16](#_Toc154010777)

[IV.1. Synthesis of PU based on HMDI and BDO (**PU**).	16](#_Toc154010778)

[V.	Polyhydroxyurethane (PHU) modification.	17](#_Toc154010779)

[V.1. General procedure for PHU modification.	17](#_Toc154010780)

[V.1.1. **PHU_1_** modified with butyraldehyde (**PHU_1_-Bu**).	17](#_Toc154010781)

[V.1.2. **PHU_1_** modified with heptaldehyde (**PHU_1_-Hept**).	20](#_Toc154010782)

[V.1.3. **PHU_1_** modified with 3,3,4,4,5,5,6,6,7,7,7-undecafluoroheptanal (**PHU_1_-FHept**).	21](#_Toc154010783)

[V.1.4. **PHU_1_** modified with benzaldehyde (**PHU_1_-Ph**).	24](#_Toc154010784)

[V.1.5. **PHU_1_** modified with 4-formylbenzonitrile (**PHU_1_-ArCN**).	25](#_Toc154010785)

[V.1.6. **PHU_1_** modified with 4-trifluoromethylbenzaldehyde (**PHU_1_-ArCF_3_**).	27](#_Toc154010786)

[V.1.7. **PHU_1_** modified with 3-phenylpropionaldehyde (**PHU_1_-PrPh**).	30](#_Toc154010787)

[V.1.8. **PHU_2_** modified with 3-phenylpropionaldehyde (**PHU_2_-PrPh**).	32](#_Toc154010788)

[V.2. Loadings for modification of PHU_1_ and PHU_2_ with various aldehydes.	34](#_Toc154010789)

[V.3. Solubility tests of PHU_1_, PHU_1_-Bu and PHU_1_-PrPh.	35](#_Toc154010790)

[V.4. Determination of the extent of modification for modified PHUs.	36](#_Toc154010791)

[V.4.1. Method 1 (by ^1^H NMR)	36](#_Toc154010792)

[V.4.2. Method 2 (by ^19^F NMR)	37](#_Toc154010793)

[V.5. Determination of regioregularity and microstructure of PHU_1_ using NMR	39](#_Toc154010794)

[V.6. Determination of crystallinity degree in polymers.	40](#_Toc154010795)

[**B.**	**Discussion and analysis**	41](#_Toc154010796)

[VI.	PHU synthesis optimization.	41](#_Toc154010797)

[VII.	Modification reaction optimization.	44](#_Toc154010798)

[VIII.	DSC plots of polymers.	47](#_Toc154010799)

[IX.	DMTA plots of polymers.	57](#_Toc154010800)

[X.	Representative WAXD plots for qualitative analysis	58](#_Toc154010801)

[XI.	Water adsorption.	60](#_Toc154010802)

[XII.	Storage moduli (G’) of polymers at different humidity levels (via torsional rheometry).	61](#_Toc154010803)

[XIII.	^1^H NMR spectra and GPC plots of selected modified PHUs	64](#_Toc154010804)

[XIV. Tensile testing	67](#_Toc154010805)

### A. **Experimental part**

# I. Materials and methods

## I.1. Materials

***Reagents****: meso*-Erythritol (>99%, TCI Chemicals), diphenyl carbonate (99%, Sigma-Aldrich), zinc (II) acetate dihydrate (≥99.0%, Sigma-Aldrich), 4-vinylcyclohexene dioxide (mixture of isomers, ≥96.0%, Sigma-Aldrich), triethylamine (≥99%, Sigma-Aldrich), lithium bis(trifluoromethanesulfonyl)imide (LiTFSI, 99.9%, Solvionic), p-toluenesulfonic acid monohydrate (≥98%, Carl Roth), dibutyltin (II) dilaurate (98%, Sigma-Aldrich), butyraldehyde (>98%, TCI Chemicals), heptanal (≥95%, Sigma-Aldrich), 4-formylbenzonitrile (>98%, TCI Chemicals), 4-trifluoromethyl aldehyde (>98%, TCI Chemicals), benzaldehyde (≥99%, Sigma-Aldrich), 3-phenylpropionaldehyde (≥95%, Sigma-Aldrich), hydrochloric acid (37wt.% in water, Carl Roth), trifluoromethane sulfonic acid (98%, Sigma Aldrich), tetrafluoroboric acid (48wt.% in water, Sigma Aldrich), sulfuric acid (>95%, Fisher Scientific) and Amberlyst® 15 hydrogen form (Sigma Aldrich) were used as received. Carbon dioxide (CO_2_, N27, Air Liquide) was used without additional purification.

Hexamethylene diamine (HMDA, 98%, Sigma-Aldrich) was purified by vacuum distillation over NaOH pellet and was kept under argon prior to use. Lithium bromide (>95%, Acros Organics) was dried at 130°C/0.5 mbar overnight in B-585 oven (Buchi Glass Drying Oven, Switzerland) filled with P_2_O_5_ and then was transferred under vacuum inside an argon-filled glovebox (MBRAUN MB-Labstar, H_2_O and O_2_ content <0.5 ppm). Butanediol (99%, Sigma-Aldrich) was purified by distillation over calcium hydride (CaH_2_). Hexamethylene diisocyanate (HMDI) (≥99%, Sigma-Aldrich) was dried at 25°C/0.5 mbar for 5 h and stored in an argon glovebox. 3,3,4,4,5,5,6,6,7,7,7-Undecafluoroheptanal (99%, P&M Invest) was distilled over concentrated sulfonic acid (H_2_SO_4_, 2-3 drops) in vacuum prior to use. Anhydrous magnesium sulfate was prepared in-house from MgSO4·7H_2_O (MgSO_4_, >99%, Aldrich) by heating a saturated aqueous solution of magnesium sulfate in a stainless steel pan (~500 mL) using a hot plate (Severin 1500 W) set to maximum heating. Following evaporation of all liquid water, heating was continued for another ~6-8 h (T > 200^o^C) to obtain an anhydrous cake. The cake was allowed to cool to ~70 ◦C, broken into ~1 cm sized pieces, then stored in a sealed glass jar prior to use.

***Solvents***: acetone (99.6%, Acros Organics), dichloromethane (>99.5%, Acros Organics), N,N-dimethylformamide (DMF, 99.8%, extra dry over molecular sieves, Acros Organics), dimethyl sulfoxide (DMSO, 99.7%, extra dry, Acros Organics), 1-methyl-2-pyrrolidinone (NMP, 99.5%, extra dry, Acros Organics), tetrahydrofuran (THF, 99.6%, Acros Organics) and diethyl ether (99+%, Acros Organics) were used as received*.* Ultrapure deionized water was obtained using Sartorius Arium^®^ Comfort smart station.

***NMR solvents and standards***: dimethyl sulfoxide-d_6_ (DMSO-d_6_, 99.9 atom%D, Sigma-Aldrich) and chloroform-d (CDCl_3_, 99.8 atom%D, Sigma-Aldrich) were dried over 4Å molecular sieves. Hexafluorobenzene (C_6_F_6_, 99%, Acros Organics) was used as received.

## I.2. Methods

***I.2.1. Nuclear magnetic resonance (NMR)*** spectra were recorded on Avance III HD 600MHz (Bruker) spectrometer (^1^H NMR at 600 MHz, ^13^C NMR at 151 MHz and ^19^F at 565 MHz) at 25°C (unless stated otherwise) in the indicated deuterated solvent and are listed in ppm. The signals corresponding to the residual protons and carbons of the deuterated solvent were used as an internal standard for ^1^H and ^13^C NMR, respectively. C_6_F_6_ (-164.9 ppm) was utilized as an external standard for ^19^F NMR. Traces of common non-deuterated solvents and impurities were identified according to *Fulmer et al.^[1]^*

***I.2.2. Size exclusion chromatography (SEC) / gel permeation chromatography (GPC)*** was used to determine the number-average molecular weights (*M*_n(GPC)_) and *M*_w_/*M*_n_ ratios. The study was performed on a 1200 Infinity gel permeation chromatograph (Agilent Technologies, USA) equipped with PLgel 5μm MIXED-D column (Agilent Technologies, USA), PLgel 5μm (Agilent Technologies) pre-column and an integrated refractive index detector. The system was operated at 50°C and 1.0 mL/min flow using 0.1 M Li(CF_3_SO_2_)_2_N (LiTFSI) solution in DMF as an eluent. Poly(methyl methacrylate) standards (EasiVial PM, Agilent Technologies, *M*_p_ = 550 - 1558×10^3^) were used to perform calibration.

***I.2.3. Thermal gravimetric analysis (TGA)*** was carried out in air on a TGA2 STARe System (Mettler Toledo) applying a heating rate of 5°C/min. The onset weight loss temperature (T_onset_) was determined as the point in the TGA curve at which a significant deviation from the horizontal was observed. The resulting temperature was then rounded to the nearest 5°C.

***I.2.4. Differential Scanning Calorimetry (DSC)*** was performed on a DSC 300 Caliris Select differential calorimeter (NETZSCH) in the range of -100 to 200°C for EDC, -130 to 180°C for 4VCHBC monomers and between -80 to 180°C for PHUs (to 210°C for the PU control). Two heating-cooling cycles with a heating rate of 5°C/min were carried out for each sample under N_2_ atmosphere. Both the glass transition (T_g_) and the melting temperatures (T_m_) were determined from the heating curve, while crystallization temperature (T_cr_) was identified from the second cooling curve. For simplicity T_m_ and T_cr_ were taken from the peak maximum.

***I.2.5. Crystallinity***

PHU powders or pieces of bars were dried at 50°C/0.1 mbar for 12 h in a B-585 oven (Buchi Glass Drying Oven) filled with P_2_O_5_ and then clamped into the DSC pellet. The analysis was performed on a DSC 300 Caliris Select differential calorimeter (NETZSCH) in the range of -80 to 180°C for PHUs (to 210°C for the PU control) with a heating rate of 10°C/min in accordance with the procedure described by *Kajiyama et al.^[2]^*

***I.2.6. Water contact angle (CA)*** measurements were performed on thin polymer films using the sessile drop method on a Contact Angle System OCA (Apollo Instruments) at 25°C and atmospheric pressure. A 10 µL drop of ultrapure deionized water was released through a motor-driven syringe onto the surface of the tested PHU film. The photo image of the drop was acquired after 15 seconds after the drop placement by a numerical camera and transmitted to a computer workstation to calculate the contact angle. The contact angles were calculated using SCA 20 v.2 software using automatic profile analysis protocol. Each reported value is an average of at least five independent measurements. Polymer films were casted on clean microscope glass slides from 0.1g/mL (PHU_1_) or 0.15g/mL (modified PHU_1_ polymers) solutions in DMF filtered preliminarily through 0.2 μm syringe filter and allowed to slowly evaporate at 80°C on a heating plate. The obtained thin films were dried at 80°C/0.1 mbar for 12 h. After cooling down in vacuum to RT films were directly applied for CA analysis to prevent the adsorption of atmospheric moisture.

***I.2.7. Thermomechanical analysis and water adsorption studies***

***Sample preparation.*** Polymer bars (typical length × width × thickness = 20 × 5 × 1.5 mm) were prepared using vacuum compression molding MeltPrep (MeltPrep GmbH) machine. Polymer powder (~ 0.15 g) was loaded in a mold and heated for 10 minutes at 90°C for amorphous samples or at 140°C for crystalline polymers under the vacuum of 0.1 mbar. Afterwards the mold was allowed to cool down to room temperature, opened and the polymer bar was released. An exception was made for the control **PU**, which had to heated for 15 minutes at 210°C given its T_m_.

***Dynamic Mechanical Thermal Analysis (DMTA)*** was carried out on polymer bars (typical length × width × thickness = 20 × 5 × 1.5 mm) with a DMA 850 “Discovery” model (TA Instruments, USA) operating in tension mode (dynamic strain: 0.05 %, pretension (preload force): 0.01 N, force track: 110%). Experiments were performed at 1 Hz frequency with a heating rate of 5°C/min from 10 to 100°C. The set up provided the storage and loss moduli (E’ and E”). The damping parameter or loss factor (tan δ) was defined as the ratio tan δ = E’’/E’. While it is common practice to determine T_α_ based on the tan δ peak, here T_α_ is derived from the loss modulus peak, in line with multiple reports specifically recommending this as the more rigorous and appropriate approach for properly defining the main relaxation temperature^[3]^.

***Rheology*** measurements were performed in torsion mode with bar geometry using an Anton Paar Physica MCR 302 rheometer equipped with a CTD 180 temperature control device and a humidity controller MHG100. Polymer samples in a form of bars (typical length × width × thickness = 20 × 5 × 1.5 mm) were stored during 72 h in the specified humidity conditions (45, 65 and 85%) before measurement and then loaded directly in the clamping system. The samples were tested in frequency sweep mode from 0.1 to 100 Hz and at an imposed 0.1% shear strain, ensuring that both moduli G′ and G″ were obtained in the linear viscoelastic regime. All measurements were carried out at 25°C and constant humidity (45, 65 or 85% RH) maintained by MHG100 humidity controller.

***Water adsorption or water uptake*** was measured for each polymer sample using bars (typical length × width × thickness = 20 × 5 × 1.5 mm) at three humidity levels: 45, 65 or 85% RH controlled according to ASTM E337-15. These bars were weighed after specified periods of time (24h, 72h, 7 days, 11 days) and water adsorption was calculated using the following formula:

$Water uptake \left( \% \right)=\frac{Weight of bar (stored at specified humidity for xh)}{Weight of bar (right after preparation)}\boldsymbol{\times}100\%$	(eq. S1)

***I.2.8. Tensile testing.***

***Sample preparation.*** Polymer dumbbells (ISO 37 type 4) were prepared using vacuum compression molding MeltPrep (MeltPrep GmbH) machine. Polymer powder (~ 0.15 g) was loaded in a mold and heated for 10 minutes at 90°C for amorphous samples or at 140°C for crystalline polymers under the vacuum of 0.1 mbar. Afterwards the mold was allowed to cool down to room temperature, opened and the polymer dumbbells was released. Then samples were conditioned for at least 24 h under at 21 ± 2 °C in closed vials to avoid excessive water uptake.

***Tensile strength and elongation at break determination.*** The quasi-static mechanical properties of the synthesized polymers were tested using an Instron 68SC-1 universal testing machine (UTM) equipped with a 1 kN loadcell and manual clamps (2716-016) operated in tension mode. Mechanical tests were performed at room temperature (21 ± 2 °C) and relative humidity of 50 ± 5 %. The crosshead speed was maintained at 2 mm/min during all experiments. To achieve statistically valid results a set of three specimens was tested for each polymeric material. The tensile modulus was measured from stress-strain plots within the Hookean region (0.1 – 0.2 % strain) with the help of Bluehill Universal (v 4.09) software.

# II. Monomer synthesis.

## II.1. Erythritol dicarbonate (EDC)

**Scheme S1.**

Adapted from *Mülhaupt* *et al* ^[4]^. *meso*-Erythritol (51.58 g, 422.4 mmol), diphenyl carbonate (190.00 g, 886.9 mmol), zinc (II) acetate dihydrate (1 mol%, 0.93 g, 4.2 mmol) and DMSO (90 mL) were placed in a 500 mL round bottom flask equipped with magnetic stirrer. Then the vacuum distillation manifold was assembled with a single 250 mL round bottom receiver flask. The vacuum was set at 3 mbar using Vacuubrand controlled membrane pump and the mixture was heated to 80°C to allow the formation of a clear solution. The temperature was slowly increased to 110°C and the distillation of phenol byproduct was observed. The reaction was continued for 1.5-2 hours 110°C/3 mbar until only solid residue remained in the flask. Afterwards the temperature was increased to 130°C, the vacuum was lowered to 0.5 mbar and the reaction allowed to continue for additional 30 minutes. The reaction flask was cooled down to 50°C, the solid residue was dissolved in 1800 mL of acetone (approx. 25 mL of acetone per 1g of product) and the mixture was refluxed for 10 minutes, whereupon it was hot-filtered through paper filter to remove undissolved residue and yellowish color. The obtained transparent solution was cooled down to RT and placed in the freezer for 12 h. The shiny-white crystalline powder was collected by filtration and dried at 100°C/1-2 mbar overnight. Yield: 64.28 g (87.4%); Melting point (DSC, 5°C/min) T_m_ = 172.1°C; Crystallization temperature (DSC, 5°C/min) T_cr_ = 127.2°C;

^1^H NMR (600 MHz, DMSO-d_6_, δ): 5.17 – 5.10 (m, 2H), 4.65 – 4.57 (m, 2H), 4.43 – 4.36 (m, 2H).

Reference spectra

This work

**Figure S1.** ^1^H NMR (600 MHz) spectrum of EDC monomer in DMSO-d_6_. Reference spectra of EDC and TDC adopted from^[5]^.

**Figure S2.** DSC plot of EDC monomer.

## II.2. 4-Vinylcyclohexene dicarbonate (4VCHBC)

**Scheme S2.**

Synthesis of 4VCCHBC was performed in a 500 mL Büchiglasuster Polyclave glass high-pressure reactor attached to the Schlenk line. Anhydrous lithium bromide (10 mol%, 3.098 g, 35.7 mmol) was weighed inside an argon filled glovebox and loaded in the Schlenk flask. The flask was removed from the glovebox, the 80 mL of anhydrous DMF were injected via syringe and stirred for 3 h until the formation of a clear solution.

The glass reactor was vacuumed for 10 minutes and then filled with argon. The LiBr (catalyst) solution in DMF was firstly injected via syringe through the inlet valve equipped with a septum. Then the 4-vinylcyclohexene dioxide (45.9 mL (d=1.09g/mL), 50 g, 356.7 mmol) was added to the reactor via syringe injection. Finally, 140 mL of anhydrous DMF were added to the reactor from the bottle with septum using a cannula. The reaction mixture was stirred for 15 minutes until the formation of the clear solution. Then the CO_2_ bottle was attached to the reactor through a high-pressure vacuum tube, the reactor was evacuated for 5 min to remove the argon atmosphere and afterwards was pressurized with 1 bar of gaseous CO_2_. The temperature was increased to 80°C and the pressure of CO_2_ was adjusted to 2 bar. The stirring was continued at 80°C and 2 bar of CO_2_ pressure for 60 h. After colling down to RT the reactor was depressurized, the reaction mixture was poured out of the reactor and the DMF was removed on rotary evaporator. The crude product was redissolved in 400 mL of dichloromethane and the resulting solution was washed with distilled water (3 × 100 mL) and dried over anhydrous magnesium sulfate. MgSO_4_ was filtered off and the solution was concentrated to 80 mL on rotary evaporator. The final product was isolated by precipitation of concentrated dichloromethane solution into 500 mL of diethyl ether. The resulting yellow oil was dried at 80°C/0.6 mbar for 12 h to provide product in a form of transparent dark yellow viscous honey-like oil. Yield: 56.8 g (69.8 %); T_g_ (DSC, 5°C/min) = -10°C;

**Figure S3.** Büchi glass high-pressure reactor with attached Huber thermostat.

 ^1^H NMR (600 MHz, DMSO-d_6_, δ): 4.97 – 4.82 (m, 2H, -CH- [5,6]), 4.71 – 4.61 (m, 1H, -CH- [3]), 4.59 – 4.28 (m, 2H, -CH_2_- [4]), 2.22 – 1.98 (m, 2H), 1.97 – 1.75 (m, 1H, -CH- [2]), 1.72 – 1.47 (m, 2H), 1.33 – 0.90 (m, 2H);

^13^C NMR (151 MHz, DMSO-d_6_, δ): 154.62 (9C, 10C), 79.27 (3C), 79.04 (3C), [75.42, 75.18, 74.60] (5C, 6C), 67.49 (4C), 67.34 (4C), 35.38 (2C), 35.22 (2C), 32.54 (2C), [28.27, 28.03, 26.07, 25.24, 24.33, 19.82, 19.33] (1C, 7C, 8C).

Anal. calcd. for C_10_H_12_O_6_ (228.2): C, 52.63%; H, 5.30%. Found: C, 52.32%; H, 5.41%.

**Figure S4.** ^1^H NMR spectrum of 4VCHBC monomer in DMSO-d_6_.

**Figure S5.** ^13^C NMR spectrum of 4VCHBC monomer in DMSO-d_6_.

**Figure S6.** ^1^H-^13^C HSQC spectrum of 4VCHBC monomer in DMSO-d_6_. Blue cross-peaks represent -CH_2_- interactions and red cross-peaks represent -CH- interactions.

**Figure S7.** ^1^H-^13^C HMBC spectrum of 4VCHBC monomer in DMSO-d_6_.

**Figure S8.** DSC plot of 4VCHBC monomer.

# III. Polyhydroxyurethane (PHU) synthesis.

III.1. Synthesis of PHU based on EDC and HMDA (**PHU_1_**).

**Scheme S3.**

A microcompounder (Figure S9) was preheated to 100°C and flushed with argon for 5 mins. The rotation of the screws in the microcompounder was initiated and the screw speed was set at 120 rpm. Erythritol dicarbonate (EDC, 8.00 g, 45.9 mmol) and hexamethylene diamine (HMDA, 5.34 g, 45.9 mmol) were quickly mixed with anhydrous N-methyl-2-pyrrloidone (NMP, 30 wt.%, 4.00 g, 3.90 mL) to form a paste and injected to microcompounder via the funnel. The funnel was removed, the injection inlet was sealed with the stainless-steel cap and the reaction was continued at 100°C for 2.5 h under inert atmosphere with a screw speed of 120 rpm.

**Figure S9.** Schematic representation (**A**) and photo (**B**) of twin-screw microcompounder Xplore MC15, that was used for reactive extrusion synthesis of PHU_1_ and PHU_2_ polymers.

**(A)**

**(B)**

Following completion of the reaction the path to the outlet die at the bottom of the microcompounder was opened and the viscous reaction mass was collected in a Teflon cup. The remaining mass was scraped out of the heating chamber and added to the batch after the microcompounder was opened. The combined mass was dissolved in 50 mL of NMP and precipitated into an excess (300-350 mL) of acetone. The PHU_1_ yellow powder was collected by filtration, extensively washed with acetone and dried at 80°C/1 mbar overnight. Yield: 12.48 g (93 %); M_n_ (GPC) = 9300-16400 g·mol^–1^, M_w_/M_n_ = 4.64-9.06; T_g_ (DSC, 5°C/min) = 50°C, T_g_ (DSC, 10°C/min) = 58°C, T_m1_ (DSC, 5°C/min) = 97°C, T_m2_ (DSC, 5°C/min) = 130°C, T_m1_ (DSC, 10°C/min) = 104°C, T_m2_ (DSC, 10°C/min) = 134°C; T_onset_ (TGA, 5°C/min) = 185°C; Anal. calcd. for C_12_H_22_N_2_O_6_ (290.316): C, 49.65%; H, 7.64%; N, 9.65%. Found: C, 50.03%; H, 7.76%; N, 9.31%;

^1^H NMR (600 MHz, DMSO-d_6_, δ): 7.16 – 6.64 (m, 2H), 5.30 – 3.46 (m, 7H), 2.94 (s, 4H), 1.37 (s, 4H), 1.22 (s, 4H);

^13^C NMR (151 MHz, DMSO-d_6_, δ): 156.56, 156.34, 156.18, 156.01, 75.02, 73.37, 72.42, 70.96, 70.15, 69.98, 67.97, 66.16, 65.83, 65.50, 63.10, 62.74, 60.13, 59.87, 40.25, 30.72, 29.46, 26.04.

**Figure S10.** ^1^H NMR spectrum of **PHU_1_** in DMSO-d_6_. Signal at 5.71 ppm was assigned to urea moieties using literature reference^[6]^.

**Figure S11.** ^13^C NMR spectrum of **PHU_1_** in DMSO-d_6_.

**Figure S12.** ^1^H NMR spectrum of **PHU_1_** in DMSO-d_6_ with 10 μL of D_2_O along with some assignments of EDC-derived moiety.

c2

c’2

b3

c1

b1

a1

b4

b2

a2

c’1

b’4

u1

u2

**Figure S13.** HSQC spectrum of the EDC-derived region of **PHU_1_** (blue – CH_2_, red – CH, CH_3_). Groups of cross-peaks for specific moieties are indicated with orange (CH_2_OH), red (CH_2_OC(=O)N), green (CH(OH)) and blue (CHOC(=O)N) circles.

b3

c2,b’2

c’2

u2

a2,b’3

b2

a1

b1

b’4,c1

u1

b4,c’1

c2

c’2

b3

b1

a1

b4

b2

a2

c’1

b’4

u1

u2

b3-1’

b3-2

b3-4

b2-3

b2-1’

b2-1”

b1-3

a1-2

b2-1”

b4-3

b4-2

c’1-2

c1-2

c1-1”

c1-1’

b3-1”

a2-1’

a2-1’’

**Figure S14.** HSQC (lightblue: CH ; red: CH_2_) and HSQC-TOCSY (blue) spectra of **PHU_1_** with assignments. The strategy used for assigning the NMR signals was adapted from the one used in previous work.^[7]^ Letters **a**, **b**, **c** represent regioisomers; the letter **u** is used to represent unsubstituted chain ends; ‘ is used to indicate a stereoisomer while ‘ and “ are used to distinguish resonances belonging to diastereotopic ^1^H resonances.

## III.2. Synthesis of PHU based on 4VCHBC and HMDA (**PHU_2_**).

**Scheme S4.**

**PHU_2_** was synthesized similarly to **PHU_1_** (SI, III.1) with the exception that the reaction of 4-vinylcycloxene dicarbonate (4VCHBC, 8.00 g, 35.1 mmol) and HMDA (4.07 g, 35.1 mmol) in the presence of anhydrous NMP (30 wt.%, 3.62g, 3.50 ml) was conducted at 100°C for 4.5 h. Yield: 8.7 g (72.1%); M_n_ (GPC) = 4800-6000 g·mol^–1^, M_w_/M_n_ = 2.07-2.33; T_g_ (DSC, 10°C/min) = 69°C; T_onset_ (TGA, 5°C/min) = 195°C;

^1^H NMR (600 MHz, DMSO-d_6_, δ): 7.14 – 6.57 (m, 2H, -NH-), 4.97 – 3.70 (m, 5H), 3.45 (m, 2H), 2.93 (s, 4H), 2.03 – 1.42 (m, 5H), 1.36 (s, 5H), 1.23 (s, 5H), 1.19 – 1.0 (m, 1H);

^13^C NMR (151 MHz, DMSO, δ): 156.35, 155.90, 77.09, 74.28, 71.44, 69.28, 60.68, 56.88, 40.17, 40.06, 30.67, 29.43, 29.39, 25.99.

***Note:*** *the two singlets at 1.36 ppm and 1.23 ppm corresponding to -CH_2_- units of the amine fragment overlap with signals of the cycloaliphatic unit of 4VCHBC fragment, resulting in higher integrations than expected.*

**Figure S15.** ^1^H NMR spectrum of **PHU_2_** in DMSO-d_6_.

**Figure S16.** ^13^C NMR spectrum of **PHU_2_** in DMSO-d_6_.

# IV. Polyurethane (PU) synthesis.

## IV.1. Synthesis of PU based on HMDI and BDO (**PU**).

**Scheme S5.**

Hexamethylene diisocyanate (HMDI) (5.55 g, 33 mmol) was weighed into a 100 mL round bottom flask with magnetic stirring bar inside the argon filled glovebox. The flask was closed with rubber septum, taken out of the glovebox and an anhydrous DMSO (34 mL) was injected via syringe upon stirring, whereupon 1,4-butanediol (2.97 g, 33 mmol) and dibutyltin (II) dilaurate (2.5 mol.%, 0.52 g, 0.83 mmol) were added consecutively to the flask using syringes. The reaction mixture was vigorously stirred during the addition of all compounds and the rapid increase in temperature up to 45-50°C was observed after the addition of the tin catalyst. After the initial exotherm the reaction flask was placed into the oil bath preheated to 60°C and the reaction was continued for 19 h. The highly viscous milky-white mass was cooled to room temperature and the polymer was isolated by precipitation into the excess of acetone. Polymer was collected by filtration, thoroughly washed with ethanol, water, and acetone and dried at 80°C/1 mbar overnight to give shiny-white powder. Yield: 6.72 g (91.6%); T_g_ (DSC, 10°C/min) = 45°C, T_m_ (DSC, 5°C/min) = 187°C, T_m_ (DSC, 5°C/min) = 188°C; T_onset_ (TGA, 5°C/min) = 200°C.

# V. Polyhydroxyurethane (PHU) modification.

## V.1. General procedure for PHU modification.

**PHU_1_-Bu**, **PHU_1_-Hept**, **PHU_1_-FHept**, **PHU_1_-PrPh, PHU_1_-Ph**, **PHU_1_-ArCF_3_** and **PHU_1_-ArCN** were prepared via post-synthetic modification of PHU_1_. While the detailed loadings for the synthesis of these polymers are presented in Table S1, the modification procedure for **PHU_1_-Bu** is provided as an example.

### V.1.1. **PHU_1_** modified with butyraldehyde (**PHU_1_-Bu**).

**Scheme S6.**

**PHU_1_** (10.00 g, 34.48 mmol) was placed in 250 ml round bottom Schlenk flask equipped with a magnetic stirrer. The flask was closed with rubber septum, evacuated, filled with argon and 80 mL of anhydrous NMP were injected via syringe. **PHU_1_** was further dissolved on stirring for 1 h at 70°C. The solution of p-toluenesulfonic acid monohydrate (catalyst, 2.49 g, 13.1 mmol) in 5 mL of anhydrous NMP was then added via syringe to the **PHU_1_** solution and stirred for 5 min, whereupon butyraldehyde (14.00 g, 194.14 mmol) was injected in one shot. The obtained solution was stirred at 70°C for 72 h. After completion of the reaction triethylamine (3 mL) was added to quench the catalyst, the stirring was continued for 1 h while the flask was cooling down to room temperature. The obtained polymer solution was precipitated into the excess of water. Solvents were removed by decantation and **PHU_1_-Bu** was dried at 60°C/1 mbar for 1 h. Afterwards it was redissolved in tetrahydrofuran and precipitated into the excess of diethyl ether. To ensure the maximum purity of the **PHU_1_-Bu** sample before the studies it was additionally purified by dialysis in acetone:water = 9:1 mixture using Spectrapor^®^ 6 (1 kDa) dialysis membranes. The acetone was removed on rotary evaporator and **PHU_1_-Bu** in a form of orange to light brown powder was dried at 80°C/1 mbar for 12 h. Yield: 9.4 g (79.2 %); T_g_ (DSC, 5°C/min) = 41°C, T_g_ (DSC, 10°C/min) = 44°C, T_m_ (DSC, 10°C/min) = 108°C; T_onset_ (TGA, 5°C/min) = 185°C; Anal. calcd. for C_15.32_H_26.98_N_2_O_6_ (335.212): C, 54.89%; H, 8.11%; N, 8.36%. Found: C, 55.02%; H, 8.0%; N, 8.2%;

Modification degree: 84%, calculated from ^1^H NMR using ratio of signals -CH_3_ (0.91 – 0.81 ppm)/-CH_2_- (2.94 ppm), see section V.3.1.;

^1^H NMR (600 MHz, DMSO-d_6_, δ): 7.28 – 6.65 (m, 2H), 5.26 – 3.37 (m, 7H), 2.94 (s, 4H), 1.62 – 1.25 (m, 8H), 1.22 (s, 4H), 0.91 – 0.81 (m, 3H);

^13^C NMR (151 MHz, DMSO, δ): 156.50, 155.90, 155.77, 154.90, 104.01, 103.22, 100.79, 100.64, 79.71, 76.78, 74.93, 74.81, 70.25, 69.94, 67.34, 65.79, 63.37, 62.71, 61.91, 60.91, 40.76, 40.21, 40.06, 36.15, 35.98, 35.80, 35.61, 35.23, 30.02, 29.41, 29.32, 29.17, 25.99, 25.95, 25.91, 24.69, 24.43, 17.07, 17.02, 16.96, 16.92, 16.84, 16.73, 14.32, 13.95, 13.88, 13.81, 13.77.

**Figure S17.** ^1^H NMR spectrum of **PHU_1_-Bu** in DMSO-d_6_.

**Figure S18.** ^13^C NMR spectrum of **PHU_1_-Bu** in DMSO-d_6_.

**Figure S19.** Superposition of HSQC (red (CH & CH_3_) and black (CH_2_)) and HMBC (blue) spectra of **PHU_1_-Bu** in DMSO-d_6_.

Fig. S20

**Figure S20**. Superposition of HSQC (CH (red)) and HMBC (blue) spectra of **PHU_1_-Bu** showing correlations between the acetal carbons and the neigbouring CH_2_ protons of butyl chain. 6 acetal resonances can be distinguished. Zoom of the selected region from Fig. S15.

### V.1.2. **PHU_1_** modified with heptaldehyde (**PHU_1_-Hept**).

**Scheme S7.**

Yield: 6.63 g (52.4 %); T_g_ (DSC, 5°C/min) = 33°C, T_g_ (DSC, 10°C/min) = 36°C, T_m_ (DSC, 10°C/min) = 111°C; T_onset_ (TGA, 5°C/min) = 215°C; Anal. calcd. for C_17.74_H_31.84_N_2_O_6_ (369.178): C, 57.72%; H, 8.69%; N, 7.59%. Found: C, 56.29%; H, 8.14%; N, 7.36%;

Modification degree: 84%. calculated from ^1^H NMR using ratio of signals -CH_3_ (0.92 – 0.81 ppm)/-CH_2_- (2.94) ppm, see section V.3.1.;

^1^H NMR (600 MHz, DMSO-d_6_, δ): 7.26 – 6.67 (m, 2H, -NH-), 5.24 – 3.40 (m, 7H), 2.94 (s, 4H), 1.65 – 1.41 (m, 2H), 1.37 (s, 4H), 1.22 (m, 10H), 0.92 – 0.81 (m, 3H);

^13^C NMR (151 MHz, DMSO-d_6_, δ): 158.07, 156.49, 155.75, 154.84, 103.38, 100.98, 95.17, 76.74, 74.92, 69.93, 65.78, 62.68, 61.90, 40.21, 40.06, 33.54, 31.19, 31.17, 29.33, 28.60, 28.49, 25.97, 23.56, 23.31, 22.00, 21.94, 13.89.

**Figure S21.** ^1^H NMR spectrum of **PHU_1_-Hept** in DMSO-d_6_.

**Figure S22.** ^13^C NMR spectrum of **PHU_1_-Hept** in DMSO-d_6_.

### V.1.3. **PHU_1_** modified with 3,3,4,4,5,5,6,6,7,7,7-undecafluoroheptanal (**PHU_1_-FHept**).

**Scheme S8.**

**PHU_1_-FHept** was synthesized according to the general procedure with some differences in purification. After synthesis **PHU_1_-FHept** was precipitated in the excess of water, solvents were decanted, the residual polymer was dried on air and then washed with n-hexane (2 × 30 mL). The residue was additionally purified by dialysis against acetone:water=9:1 mixture using Spectrapor^®^ 6 (1 kDa) dialysis membrane. The acetone was removed on rotary evaporator and polymer in a form of sticky red paste was dried at 80°C/1 mbar for 12 h.

It was found by NMR analysis that the obtained **PHU_1_-FHept** sample contains oligomerized 3,3,4,4,5,5,6,6,7,7,7-undecafluoroheptanal as impurity. Thus, **PHU_1_-FHept** was further purified by Soxhlet extraction with diethyl ether for 12 h. The residual solid was dried at 80°C/1 mbar for 12 h to give a red powder. Yield: 2.85 g (52.7 %); T_g_ (DSC, 5°C/min) = 34°C, T_g_ (DSC, 10°C/min) = 42°C, T_m_ (DSC, 10°C/min) = 111°C; T_onset_ (TGA, 5°C/min) = 155°C;

Modification degree: 35%. Determined using ^19^F NMR spectrum with hexafluorobenzene as external standard (23 mg of polymer and 12.1 mg of hexafluorobenzene) using the ratio of signal at -82.69 ppm (-CF_3_) to signal at -164.9 ppm (C_6_F_6_), see section V.3.2.;

^1^H NMR (600 MHz, DMSO-d_6_, δ): 7.23 – 6.63 (m, 2H), 5.52 – 3.40 (m, 10H), 2.94 (s, 4H), 1.37 (s, 4H), 1.23 (s, 5H).

^13^C NMR (151 MHz, DMSO, δ): 156.51, 155.85, 154.87, 76.76, 69.94, 69.03, 67.93, 67.71, 65.79, 65.46, 65.06, 64.14, 59.81, 45.78, 40.75, 40.20, 40.05, 29.42, 26.01, 25.74, 8.62.

^19^F NMR (565 MHz, DMSO-d_6_, δ): -82.69, -108.53, -117.20, -123.57, -124.27, -124.56, -124.92, -128.24.

**Figure S23.** ^1^H NMR spectrum of **PHU_1_-FHept** in DMSO-d_6_.

**Figure S24.** ^13^C NMR spectrum of **PHU_1_-FHept** in DMSO-d_6_.

**Figure S25.** ^19^F NMR spectrum of **PHU_1_-FHept** in DMSO-d_6_.

### V.1.4. **PHU_1_** modified with benzaldehyde (**PHU_1_-Ph**).

**Scheme S9.**

**PHU_1_-Ph** was synthesized and purified according to general procedure. The polymer was additionally purified by Soxhlet extraction with diethyl ether overnight. Then the polymer was collected and dried at 80°C in vacuum (1-2 mbar) overnight. Yield: 7.65 g (58.7 %); T_g_ (DSC, 5°C/min) = 43°C, T_m_ (DSC, 5°C/min) = 94°C, T_g_ (DSC, 10°C/min) = 50°C, T_m_ (DSC, 10°C/min) = 100°C; T_onset_ (TGA, 5°C/min) = 205°C;

Modification degree: 43%. calculated from ^1^H NMR using ratio of signals corresponding to Ph group at 7.46 – 7.28 ppm and to -CH_2_- at 2.95 ppm, see section V.3.1;

^1^H NMR (600 MHz, DMSO-d_6_, δ): 7.46 – 7.28 (m, 2H), 7.28 – 6.67 (m, 2H), 6.13 – 3.47 (m, 8H), 2.95 (dd, *J* = 14.4, 6.9 Hz, 4H), 1.44 – 1.31 (s, 4H), 1.23 (s, 4H).

^13^C NMR (151 MHz, DMSO-d_6_, δ): 156.50, 155.89, 155.78, 154.91, 137.52, 136.85, 136.19, 130.45, 129.50, 128.93, 128.61, 128.26, 128.19, 128.05, 127.96, 127.77, 127.04, 126.85, 126.53, 126.33, 126.23, 102.67, 100.12, 100.01, 80.17, 77.22, 76.75, 75.48, 75.32, 70.66, 69.94, 67.93, 67.71, 67.02, 65.79, 65.45, 65.06, 64.91, 64.15, 62.64, 61.95, 61.54, 60.89, 60.48, 59.82, 45.75, 40.78, 40.20, 40.06, 30.45, 29.42, 29.33, 29.19, 26.52, 26.00, 25.12, 15.16.

**Figure S26.** ^1^H NMR spectrum of **PHU_1_-Ph** in DMSO-d_6_.

**Figure S27.** ^13^C NMR spectrum of **PHU_1_-Ph** in DMSO-d_6_.

### V.1.5. **PHU_1_** modified with 4-formylbenzonitrile (**PHU_1_-ArCN**).

**Scheme S10.**

**PHU_1_-ArCN** was synthesized and purified according to general procedure. The polymer was additionally purified by Soxhlet extraction with diethyl ether overnight. Then the polymer was collected and dried at 80°C in vacuum (1-2 mbar) overnight. Yield: 4.98 g (42.0 %); T_g_ (DSC, 5°C/min) = 52°C, T_m_ (DSC, 5°C/min) = 115°C, T_g_ (DSC, 10°C/min) = 55°C, T_m_ (DSC, 10°C/min) = 119°C; T_onset_ (TGA, 5°C/min) = 175°C;

Modification degree: 51%. Calculated from ^1^H NMR using ratio of signals corresponding to ArCN group at 7.98 – 7.49 ppm and to -CH_2_- at 2.94 ppm, see section V.3.1;

^1^H NMR (600 MHz, DMSO-d_6_, δ): 7.98 – 7.49 (m, 2H), 7.45 – 6.67 (m, 2H), 6.07 – 3.45 (m, 8H), 2.94 (s, 4H), 1.37 (s, 4H), 1.22 (s, 4H).

^13^C NMR (151 MHz, DMSO-d_6_, δ): 156.50, 155.72, 151.00, 141.93, 132.66, 132.35, 132.29, 132.20, 132.16, 132.10, 128.38, 127.95, 127.74, 127.43, 127.19, 127.03, 126.73, 118.92, 118.54, 112.26, 109.61, 102.17, 101.42, 75.79, 69.93, 68.23, 67.92, 67.14, 65.78, 61.82, 59.82, 52.31, 40.19, 40.05, 30.33, 30.01, 29.41, 29.32, 25.99.

**Figure S28.** ^1^H NMR spectrum of **PHU_1_-ArCN** in DMSO-d_6_.

**Figure S29.** ^13^C NMR spectrum of **PHU_1_-ArCN** in DMSO-d_6_.

### V.1.6. **PHU_1_** modified with 4-trifluoromethylbenzaldehyde (**PHU_1_-ArCF_3_**).

**Scheme S11.**

Yield: 9.3 g (60.5 %); T_g_ (DSC, 5°C/min) = 57°C, T_g_ (DSC, 10°C/min) = 59°C; T_onset_ (TGA, 5°C/min) = 195°C; Anal. calcd. for C_16.88_H_23.83_N_2_O_6_F_1.83_ (385.541): C, 52.59%; H, 6.23%; N, 6.66%. Found: C, 52.56%; H, 6.0%; N, 7.27%;

Modification degree: 61%. Calculated independently from ^1^H NMR (using ratio of signals at 7.97-7.55 ppm and the signal 3.02-2.86 ppm) and ^19^F NMR with external reference – hexafluorobenzene (17.5 mg of C_6_F_6_ and 38.0 mg of polymer, using ratio of the signal at -63.52 ppm and the signal at -164.9 ppm), see section V.3.2.;

^1^H NMR (600 MHz, DMSO-d_6_, δ): 7.96 – 7.56 (m, 2H), 7.45 – 6.67 (m, 2H), 6.25 – 5.15 (m, 1H), 5.06 – 3.48 (m, 7H), 2.95 (s, 4H), 1.36 (s, 4H), 1.23 (s, 4H).

^13^C NMR (151 MHz, DMSO-d_6_, δ): 156.52, 155.89, 155.76, 154.88, 141.64, 141.36, 130.06, 129.85, 129.59, 129.38, 128.37, 127.86, 127.61, 127.31, 127.08, 126.95, 125.18, 124.98, 123.18, 102.18, 101.58, 98.99, 98.89, 80.27, 77.32, 76.77, 76.33, 75.77, 70.68, 69.96, 67.79, 67.73, 65.80, 65.06, 63.21, 62.55, 61.87, 60.79, 60.50, 59.83, 29.41, 29.33, 29.21, 26.02, 25.99.

^19^F NMR (565 MHz, DMSO-d_6_, δ): -63.52.

**Figure S30.** ^1^H NMR spectrum of **PHU_1_-ArCF_3_** in DMSO-d_6_.

**Figure S31.** ^13^C NMR spectrum of **PHU_1_-ArCF_3_** in DMSO-d_6_. Hexafluorobenzene ^13^C NMR spectrum from Sigma was used as a reference^[8]^.

**Figure S32.** ^19^F NMR spectrum of **PHU_1_-ArCF_3_** in DMSO-d_6_.

**Figure S33.** ^1^H-^13^C HSQC spectrum of **PHU_1_-ArCF_3_** in DMSO-d_6_. Blue cross-peaks represent -CH- interactions and red cross-peaks represent -CH_2_- interactions.

**Figure S34.** ^1^H-^13^C HMBC spectrum of **PHU_1_-ArCF_3_** in DMSO-d_6_.

### V.1.7. **PHU_1_** modified with 3-phenylpropionaldehyde (**PHU_1_-PrPh**).

**Scheme S12.**

Yield: 5.42 g (38.7 %); T_g_ (DSC, 5°C/min) = 42°C, T_g_ (DSC, 10°C/min) = 46°C; T_onset_ (TGA, 5°C/min) = 225°C; Anal. calcd. for C_19.2_H_28.4_N_2_O_6_ (383.246): C, 60.17%; H, 7.47%; N, 7.31%. Found: C, 61.26%; H, 7.25%; N, 6.97%;

Modification degree: 80%. Calculated from ^1^H NMR based on the ratio of the signal at 2.63 or 1.92 – 1.68 ppm to the signal at 2.93 ppm, see section V.3.1.;

^1^H NMR (600 MHz, DMSO-d_6_, δ): 7.44 – 6.64 (m, 7H), 5.28 – 3.35 (m, 7H), 2.93 (s, 4H), 2.63 (q, *J* = 8.9 Hz, 2H), 1.92 – 1.68 (m, 2H), 1.35 (s, 4H), 1.20 (s, 4H).

^13^C NMR (151 MHz, DMSO-d_6_, δ): 156.48, 155.76, 154.86, 141.31, 141.21, 128.29, 128.21, 128.18, 128.14, 125.78, 102.68, 100.27, 76.90, 75.07, 69.93, 67.37, 65.78, 62.65, 61.88, 40.43, 40.22, 40.06, 35.36, 35.13, 30.67, 29.62, 29.31, 29.26, 25.96, 25.93.

**Figure S35.** ^1^H NMR spectrum of **PHU_1_-PrPh** in DMSO-d_6_.

**Figure S36.** ^13^C NMR (600 MHz, DMSO-d_6_) spectrum of **PHU_1_-PrPh** in DMSO-d_6_.

### V.1.8. **PHU_2_** modified with 3-phenylpropionaldehyde (**PHU_2_-PrPh**).

**Scheme S13.**

Yield: 7.45 g (72.1 %); T_g_ (DSC, 10°C/min) = 70°C; T_onset_ (TGA, 5°C/min) = 145°C;

Modification degree: 50%. Calculated from ^1^H NMR using the ratio of signals at 2.79 – 2.65 ppm to the signal at 2.94 ppm, see section V.3.1;

^1^H NMR (600 MHz, DMSO-d_6_, δ): 7.38 – 7.12 (m, 5H), 7.08 – 6.57 (m, 2H), 4.94 – 3.37 (m, 9H), 2.94 (s, 4H), 2.79 – 2.65 (m, 1H), 2.07 – 1.42 (m, 7H), 1.36 (s, 4H), 1.23 (s, 4H), 1.18 – 1.00 (m, 1H).

^13^C NMR (151 MHz, DMSO-d_6_, δ): 194.98, 156.38, 156.31, 155.78, 145.83, 141.95, 140.74, 139.16, 138.59, 137.82, 137.54, 128.85, 128.81, 128.71, 128.58, 128.37, 128.35, 128.30, 128.27, 128.24, 128.22, 128.17, 126.95, 126.85, 126.08, 125.86, 108.53, 71.44, 69.32, 60.72, 54.91, 48.87, 37.15, 36.90, 33.65, 30.63, 29.42, 28.82, 26.01.

**Figure S37.** ^1^H NMR spectrum of **PHU_2_-PrPh** in DMSO-d_6_.

**Figure S38.** ^13^C NMR spectrum of **PHU_2_-PrPh** in DMSO-d_6_.

## V.2. Loadings for modification of PHU_1_ and PHU_2_ with various aldehydes.

**Table S1.** Loadings for modification of PHU_1_ with various aldehydes *^a^*.

| Resulting polymer | Parent polymer (PHU_1_ or PHU_2_) | | Aldehyde | | | *p*-TSA | | NMP | NEt_3_ | |
| --- | --- | --- | --- | --- | --- | --- | --- | --- | --- | --- |
|  | mmol | g | structure | mmol | g | mmol | g | ml | mmol | ml |
| **PHU_1_- Bu** | 34.48 | 10 |  | 194.14 | 14 | 13.1 | 2.489 | 85 | 40.8 | 3 |
| **PHU_1_- Hept** | 32.76 | 9.5 |  | 184.4 | 21.06 | 12.44 | 2.365 | 81 | 39.5 | 2.9 |
| **PHU_1_- FHept** | 9.27 | 2.69 |  | 52.17 | 16.28 | 3.52 | 0.67 | 35.5 | 12.3 | 0.9 |
| **PHU_1_- Ph** | 34.48 | 10 |  | 194.14 | 20.6 | 13.1 | 2.489 | 85 | 40.8 | 3 |
| **PHU_1_- ArCN** | 34.48 | 10 |  | 194.14 | 25.46 | 13.1 | 2.489 | 85 | 40.8 | 3 |
| **PHU_1_- ArCF_3_** | 34.48 | 10 |  | 194.14 | 33.8 | 13.1 | 2.489 | 85 | 40.8 | 3 |
| **PHU_1_- PrPh** | 34.48 | 10 |  | 194.14 | 26.05 | 13.1 | 2.489 | 85 | 40.8 | 3 |
| **PHU_2_- PrPh** | 7.9 | 22.94 |  | 129.14 | 17.33 | 8.71 | 1.656 | 65 | 32.7 | 2.4 |

*^a^* Reaction conditions: T = 70°C, τ = 72h.

## V.3. Solubility tests of PHU_1_, PHU_1_-Bu and PHU_1_-PrPh.

**Table S2.** Solubility tests of PHU_1_, PHU_1_-Bu and PHU_1_-PrPh with various aldehydes *^a^*.

| Solvent^b^ | PHU_1_ | | | PHU_1_-Bu | | | PHU_1_-PrPh | | |
| --- | --- | --- | --- | --- | --- | --- | --- | --- | --- |
|  | 25 °C | 50 °C | 80 °C | 25 °C | 50 °C | 80 °C | 25 °C | 50 °C | 80 °C |
| DMF | - | + | + | + | + | + | + | + | + |
| NMP | + | + | + | + | + | + | + | + | + |
| DMSO | - | + | + | + | + | + | + | + | + |
| DMAc | - | + | + | + | + | + | + | + | + |
| THF | - | - | n.d. | + | + | n.d. | + | + | n.d. |
| Acetone | - | - | n.d. | - | + | n.d. | - | + | n.d. |
| Acetone:water=9:1 | - | - | n.d. | + | + | n.d. | + | + | n.d. |
| DCM | - | n.d. | n.d. | - | n.d. | n.d. | - | n.d. | n.d. |
| Ethanol | - | - | n.d. | - | - | n.d. | - | - | n.d. |
| Ethyl acetate | - | - | n.d. | - | - | n.d. | - | - | n.d. |
| Toluene | - | - | - | - | - | - | - | - | - |
| Dioxane | - | - | - | - | - | - | - | - | - |
| Diethyl ether | - | n.d. | n.d. | - | n.d. | n.d. | - | n.d. | n.d. |
| Acetonitrile | - | - | n.d. | - | - | n.d. | - | - | n.d. |

*^a^* Solubility tests were conducted with approximately 0.05 g of polymer immersed in 1 ml of selected solvent in transparent vial equipped with magnetic stirrer. The resulting mixture was stirred for 15 mins at selected temperature and solubility, or non-solubility of the sample was determined visually; ^b^ DMF – dimethylformamide, NMP – N-methyl pyrrolidone, DMSO – dimethyl sulfoxide, DMAc – dimethylacetamide, DCM – dichloromethane.

## V.4. Determination of the extent of modification for modified PHUs.

### V.4.1. Method 1 (by ^1^H NMR)

This method was used for determination of modification degrees in **PHU_1_-Bu**, **PHU_1_-Hept**, **PHU_1_-Ph**, **PHU_1_-ArCN**, **PHU_1_-ArCF_3_**, **PHU_1_-PrPh** and **PHU_2_-PrPh**. The calculation of modification degree is shown by an example of **PHU_1_-Bu** analysis.

$$Mod. degree=\frac{2.54}{3}\times100\%=84\%$$

**Figure S39.** Calculations of modification degree in **PHU_1_-Bu** from ^1^H NMR (DMSO-d_6_).

The modification degree in **PHU_1_-Bu** was calculated according to the equation:

$Mod. degree=\frac{I}{n_{H}}\times100\%$	(eq. S2)

…where I is the intensity of a signal at 0.91-0.82 ppm (CH_3_ group from butyraldehyde) normalized using the signal at 3.02-2.86 ppm (-**CH_2_**-NH- group in polymer backbone) in ^1^H NMR spectrum (Figure S38) and n_H_ is the theoretical number of H atoms.

According to (eq. S2) the modification degree of **PHU_1_-Bu** can be calculated as:

$Mod. degree=\frac{2.54}{3}\times100\%=84\%$

The modification degrees for other samples were calculated in a similar manner using respective ^1^H NMR spectra.

### V.4.2. Method 2 (by ^19^F NMR)

This method was used for determination of modification degrees in **PHU_1_-FHept** and **PHU_1_-ArCF_3_**.

First, it was confirmed that signal ratios in ^1^H and ^19^F spectra are sufficiently independent from relaxation delay. Both 1 s and 10 s relaxation delays provided comparable ratios between signals for ^1^H and ^19^F spectra (Figure S39). The calculations of modification degree have been performed using ^1^H and ^19^F spectra with 10 s relaxation delay.

**(D)**

**(C)**

**(B)**

**(A)**

**Figure S40**. NMR spectra of **PHU_1_-ArCF_3_**: (**A**) ^1^H NMR spectrum with 1 s relaxation delay in DMSO-d_6_ + C_6_F_6_; (**B**) ^1^H NMR spectrum with 10 s relaxation delay in DMSO-d_6_ + C_6_F_6_; (**C**) ^19^F NMR spectrum with 1 s relaxation delay in DMSO-d_6_ + C_6_F_6_; (**D**) ^19^H NMR spectrum with 10 s relaxation delay in DMSO-d_6_ + C_6_F_6_.

The modification degree from ^19^F NMR was calculated using the ratio between signals of -CF_3_ group in polymer and of hexafluorobenzene (C_6_F_6_), as external standard, according to the equations eq. S3 – S6:

$Mod.degree= \frac{\nu_{mod}}{\nu_{mod}+\nu_{unmod}}\times100\%$	(eq. S3)

…where $\nu_{mod}$ is the number of moles of modified repeat units in polymer chain, $\nu_{unmod}$ is the number of moles of unmodified repeat units in polymer chain;

$\nu_{mod}= \frac{I}{n_{F}}\times\nu_{C_{6}F_{6}}$	(eq. S4)

…where $I$ is the intensity of a signal at -63.52 ppm (-CF_3_ group) normalized using the signal at -164.9 ppm (C_6_F_6_) in ^19^F NMR spectrum (Figure S39D), $n_{F}$ is the theoretical number of F atoms and $\nu_{C_{6}F_{6}}$ is the number of moles of hexafluorobenzene added to the NMR sample;

$\nu_{unmod}= \frac{m_{unmod}}{M_{unmod}}= \frac{{(m}_{pol}-m_{mod})}{M_{unmod}}= \frac{{(m}_{pol}-\nu_{mod}\times M_{mod})}{M_{unmod}}= \frac{{(m}_{pol}-\frac{I}{n_{F}}\times\nu_{C_{6}F_{6}}\times M_{mod})}{M_{unmod}}$	(eq. S5)

…where $m_{pol}$ is the weight of the polymer sample, $M_{mod}$ is the molecular weight of modified repeatunit, $M_{unmod}$ is the molecular weight of unmodified repeat unit;

$Mod.degree= \frac{\frac{I}{n_{F}}\times\nu_{C_{6}F_{6}}}{\frac{I}{n_{F}}\times\nu_{C_{6}F_{6}}+\frac{{(m}_{pol}-\frac{I}{n_{F}}\times\nu_{C_{6}F_{6}}\times M_{mod})}{M_{unmod}}} \times100\%$	(eq. S6)

The weight of **PHU_1_-ArCF_3_** sample was 38.0 mg and the weight of C_6_F_6_ was 17.5 mg. Thus according to (eq. S6) the modification degree is:

$Mod.degree= \frac{\frac{1.91}{3}\times\frac{17.5 mg}{186.05 \frac{g}{mol}}}{\frac{1.91}{3}\times\frac{17.5 mg}{186.05 \frac{g}{mol}}+\frac{(38 mg-\frac{1.91}{3}\times\frac{17.5 mg}{186.05\frac{g}{mol}}\times446 \frac{g}{mol})}{290 \frac{g}{mol}}} \times100\%=60.6\% \approx61\%.$

Determination of modification degree of **PHU_1_-FHept** was conducted in similar manner using respective ^19^F NMR spectrum (Figure S25).

## V.5. Determination of regioregularity and microstructure of PHU_1_ using NMR

**Figure S41**. Analysis of PHU_1_ regioregularity using ^1^H and ^13^C NMR spectra.

The ratio between three isomeric repeat units in **PHU_1_** structure was estimated independently from ^1^H and ^13^C NMR spectra (Fig. S40) using the following equations:

$\nu_{1}= \frac{I_{1}}{I_{1}+\frac{I_{2}}{2}+\frac{I_{3}}{2}}\times100\%$	(eq. S7)

$\nu_{2}= \frac{\frac{I_{2}}{2}}{I_{1}+\frac{I_{2}}{2}+\frac{I_{3}}{2}}\times100\%$	(eq. S8)

$\nu_{3}= \frac{\frac{I_{3}}{2}}{I_{1}+\frac{I_{2}}{2}+\frac{I_{3}}{2}}\times100\%$	(eq. S9)

where ν_n_ – is the molar share of corresponding repeat unit (n = 1, 2 or 3), I_1_ is the intensity of the signal at 4.015-3.95 ppm in ^1^H NMR spectrum or the signal at 65.50 ppm in ^13^C NMR both marked with orange, I_2_ is the intensity of the signal at 4.155-4.07 ppm in ^1^H NMR spectrum or the signal at 65.83 ppm in ^13^C NMR both marked with green, I_3_ is the intensity of the signal at 4.215-4.16 ppm in ^1^H NMR spectrum or the signal at 65.83 ppm in ^13^C NMR both marked with blue. Signal in unit 1 corresponds to 1 proton or carbon and signals in units 2 and 3 correspond to 2 protons or carbons each due to internal symmetry. The calculations using ^13^C spectra were performed with the assumption that NOE effects for these carbon atoms are similar since all considered signals are originating from -CH_2_- groups in a similar environment.

The ratio between primary and secondary OH groups was determined using the following equations:

${OH}_{primary}=\nu_{3}+ \frac{\nu_{1}}{2}$	(eq. S10)

${OH}_{seconday}=\nu_{2}+ \frac{\nu_{1}}{2}$	(eq. S11)

## V.6. Determination of crystallinity degree in polymers.

The degree of crystallinity in **PU** was calculated using the following equation:

$Degree of crystallinity = \frac{\Delta H_{m}}{\Delta H_{m}(100\%)}\times100\%=\frac{105.9}{109}\times100\%=56 \%$ (eq. S12)

…where $\Delta H_{m}$ is the heat of fusion for melting transition measured by DSC at a heating rate of 10°C/min and $\Delta H_{m}(100\%)$ is the heat of fusion of 100% crystalline **PU** based of HMDI and BDO which is equal to 189 J/g as reported by several authors*^[9]^*.

The degree of crystallinity for PHU_1_ and modified PHUs was calculated in a similar manner using equation (eq. S12), with the same value for the heat of fusion of the 100% crystalline sample (189 J/g)^7^ from the fully crystalline **PU** control used as mentioned above given the structural similarities of the polymers and the lack of any more appropriate reference data in the literature. Nevertheless, it is emphasized that this implies a greater level of uncertainty as far as the accuracy of these values are concerned vs. those calculated for the **PU** control.

### B. **Discussion and analysis**

# VI. PHU synthesis optimization.

The optimization of PHU synthesis was conducted by investigation of the rection between EDC and HMDA via reactive extrusion (Scheme S14).

**Scheme S14.**

In accordance with previous reports^[10]^, the rate of this reaction can be enhanced with the increase in temperature. However, at temperatures above 100°C the occurrence of side reactions was noted^8^. Thus, to ensure the highest possible efficiency of the main reaction, i.e. the formation of linear PHU, and at the same time to avoid the probability of side reactions, the temperature of 100°C was initially selected for the synthesis of **PHU_1_**.

First, the bulk extrusion process was examined for **PHU_1_** synthesis (Table S3, entries 1-3). However, significant variations in polymer molecular weights (from 4300 to 11000 g·mol^–1^), molecular weight distributions (polydispersities from 3.1 to 14.3) and visual appearance (Figure S42, 1-3) were observed during several similar trials. Such inconsistent results were caused by the inhomogeneity of the reaction mixture inside the microcompounder due to the melting point of EDC (T_m_=172°C) being above the reaction temperature. Moreover, it was previously suggested^9a^ that extensive hydrogen bonding between hydroxyl-rich PHU molecules can limit the mobility of the chain ends and thus prevents the growth of the polymer molecular weight. The next step in this work consisted of the investigation of the influence of various solubilizing and non-solubilizing additives on the molecular weight and yield of **PHU_1_** (Fig. S41). A trihexyltetradecyl-phosphonium chloride ([C_6_C_6_C_6_C_14_P]^+^Clˉ) ionic liquid was firstly examined as a non-solubilizing hydrogen bond disruptor agent varying its quantity from 5 wt. % to 50 wt.% (Table S3, entries 4-10). It was selected due to the several reasons: a) low melting point; b) known ability of the halogen anions to form numerous hydrogen bonds^[11]^; c) low vapor pressure; d) excellent lubricating ability^[12]^. It was found that the addition of [C_6_C_6_C_6_C_14_P]^+^Clˉ to the monomer mixture influenced the molecular weight of the obtained **PHU_1_** , which was passing through its maximum of 27300 g·mol^–1^ for 40 wt. % of added [C_6_C_6_C_6_C_14_P]^+^Clˉ (Table S3, entry 9). It is important to note that in spite of the increase in polymer M_n_, the observed M_w_/M_n_ ratios were found to be very broad (Table S3, entries 4,5,8,9) consistent with the inhomogeneous appearance of polymer samples (Figure S42, 4-10). The change in viscosity and solvating ability of an ionic liquid by the replacement Brˉ resulted in a decrease in polymer molecular weight (Table S3, entry 7 vs 11,12).

**Figure S42.** Structures of solubilizing and non-solubilizing additives used in **PHU_1_** synthesis.

**Figure S43.** The appearance of **PHU_1_** samples obtained in bulk (1-3) and using [C_6_C_6_C_6_C_14_P]^+^Clˉ (4-10) non-solubilizing additive.

**Figure S44.** The appearance of **PHU_1_** samples obtained using various solubilizing additives.

Table S3. Optimization of PHU_1_ synthesis^a^.

|  | **Entry** | **Additive** | **Additive,**  **wt. %** | **Yield, %** | **M_n_,** **g·mol^–1^** **^b^** | **M_w_/M_n_^b^** |
| --- | --- | --- | --- | --- | --- | --- |
| Non-solubilizing  additives | 1 | - | - | 70 | 11000 | 3.1 |
|  | 2 | - | - | 68 | 4300 | 3.9 |
|  | 3 | - | - | 71 | 5300 | 14.3 |
|  | 4 | [C_6_C_6_C_6_C_14_P]^+^Clˉ | 5 | 75 | 7400 | 10.1 |
|  | 5 | [C_6_C_6_C_6_C_14_P]^+^Clˉ | 5+5 | 73 | 13300 | 15.1 |
|  | 6 | [C_6_C_6_C_6_C_14_P]^+^Clˉ | 10 | 75 | 11400 | 6.8 |
|  | 7 | [C_6_C_6_C_6_C_14_P]^+^Clˉ | 20 | 81 | 18600 | 4 |
|  | 8 | [C_6_C_6_C_6_C_14_P]^+^Clˉ | 30 | 80 | 6900 | 10.2 |
|  | 9 | [C_6_C_6_C_6_C_14_P]^+^Clˉ | 40 | 68 | 27300 | 10.1 |
|  | 10 | [C_6_C_6_C_6_C_14_P]^+^Clˉ | 50 | 70 | 21200 | 4.5 |
|  | 11 | [C_6_C_6_C_6_C_14_P]^+^Brˉ | 20 | 73 | 4900 | 3.6 |
|  | 12 | [C_6_C_6_C_6_C_14_P]^+^N(CN)_2_ˉ | 20 | 70 | 7100 | 4.4 |
| Solubilizing  additives | 13 | DMSO | 20 | 82 | 12600 | 6.1 |
|  | 14 | DMF | 20 | 82 | 11500 | 5.9 |
|  | 15 | DMAc | 20 | 50 | 6300 | 2.8 |
|  | 16 | Sulfolane | 20 | 70 | 9300 | 5.3 |
|  | 17 | HMPA | 20 | 58 | 10100 | 2.8 |
|  | 18 | NMP | 20 | 80 | 15100 | 6.5 |
|  | 19 | NMP | 30 | 80 | 9400 | 3.9 |
|  | 20 | NMP | 40 | 80 | 7800 | 3.3 |

^a^ Synthetic conditions: T = 100°C, 2.5 h, screw rotation speed – 120 rpm; ^b^ By GPC in 0.1 M solution of LiTFSI in DMF at 50 °C with PMMA standards.

As the next step, several solubilizing additives, representing organic solvents with high boiling points and the ability to dissolve EDC and HMDA monomers were evaluated (Table S3 and Figure S41). The amount of solubilizing additive was fixed at 20 wt. %, based on the best result achieved with [C_6_C_6_C_6_C_14_P]^+^Clˉ (Table S3, entry 7). The utilization of solubilizing additives (Tables S2, entries 13-18) allowed the synthesis of **PHU_1_** samples with significantly lower M_w_/M_n_ ratios (2.8-6.5) and moderate to high molecular weights (6300-15100 g·mol^–1^). At similar M_w_/M_n_ ratios (5.9-6.5), the highest **PHU_1_** molecular weights were reached when dimethylformamide, DMF (11500 g·mol^–1^), dimethyl sulfoxide, DMSO (12600 g·mol^–1^) and N-methylpyrrolidone, NMP (15000 g·mol^–1^) were applied. The appearance of **PHU_1_** samples synthesized in the presence of solubilizing additives was found to be more uniform in comparison with the appearance of the samples obtained with nonsolubilizing additives (Figures 42 and 43). The samples obtained in dimethylacetamide (DMAc), hexamethylphosphoramide (HMPA) and N-methylpyrrolidone (NMP) displayed the greatest homogeneity (Figure S43). The increase in NMP amount from 20 and 40 wt. % resulted in a simultaneous decrease in molecular weight to 7800 g·mol^–1^ and M_w_/M_n_ ratio to 3.3 (Table S3, entries 18-20). Based on a detailed analysis of all the experiments in Table S3, it was concluded that the optimal reaction conditions for the synthesis of **PHU_1_** with highest possible molecular weight, lowest M_w_/M_n_ ratio, best homogeneity and in highest yield are as follows: 30 wt.% of NMP (solubilizing additive and hydrogen bond disruptor), 100°C and 2.5 h.

# VII. Modification reaction optimization.

The modification of **PHU_1_** polymer with butyraldehyde (Scheme S15) was conducted in solution to prevent the potential cross-linking between polymer chains due to formation of linear acetals instead of cyclic ones. *p*-Toluenesulfonic acid (*p*-TSA) was used as a catalyst due to its mild acidity (to prevent acidic hydrolysis of PHU polymer) and good solubility in organic solvents.

**Scheme S15.**

In this study four different reaction parameters such as temperature, duration, catalyst to polymer and aldehyde to polymer ratios were investigated (Table S4).

At first, the influence of temperature on polymer yield and extent of modification was studied (Table S4, entries 1-8). While all other parameters such as reaction duration (24 h), aldehyde to polymer ratio (7:1 by mol) and *p*-TSA to polymer ratio (0.38:1 by mol) were fixed, the temperature has been varied between 40 and 90°C. Figure S44A shows the temperature dependence of **PHU_1_** modification degree. The extent of modification was passing through the maximum at 70 - 75°C. At the same time polymer yield was found to be dependent on temperature to a lesser extent and was ranging from 66 to 73 % (Table S4, entries 1-8).

In a next step the influence of the reaction duration was investigated keeping the following parameters constant: temperature at 40°C, aldehyde to polymer ratio equal to 7:1 by mol and *p*-TSA to polymer ratio equal to 0.38:1 by mol (Table S4, entries 9-13). The gradual enhancement in the extent of modification was observed increasing the reaction duration from 24 to 60 h, whereupon the modification degree was reaching a plateau at ~88-90% (Figure S44B).

The effect of catalyst to polymer ratio on **PHU_1_** modification degree is demonstrated in Figure S44C. It can be concluded that the utilization of *p*-TSA as a catalyst resulted in high values (>74%) of the extent of modification (Table S4, entries 14-20). At low *p*-TSA loading (*p*-TSA to polymer – 0.09:1, Table S4, entry 14) the partial cross-linking was observed, probably due to the formation of linear acetals between two polymer chains. The 0.22:1 catalyst to polymer ratio provided the highest degree of modification reaching 87% (Table S4, entry 16).

The catalytic effect of several Bronsted acids was studied as well (Figure S45). It was shown that the water content is playing more significant role that the acidity of the catalyst. Thus, the use of *p*-TSA, CF_3_SO_3_H and H_2_SO_4_ with lower water content resulted in the preparation of **PHU_1_-Bu** with higher modification degrees (81-86%) in comparison with the utilization of aqueous HCl or HBF_4_ solutions (71 and 78%, respectively). The use of heterogeneous Amberlyst® 15 (hydrogen form) catalyst was leading to the formation of insoluble cross-linked polymer. This observation is in accordance with the literature reports on the use of heterogeneous catalysts for similar modification of poly(vinyl alcohol) ^[13]^. Moreover, as it was found previously^[14]^, exactly the presence of water traces in heterogeneous Amberlyst® 15 can lead to the unwanted cross-linking of poly(vinyl alcohol).

Finally, the influence of aldehyde to polymer ratio on polymer yield and extent of modification was investigated (Table S4, entries 21-26). As it can be seen from Figure S44D the modification degree was gradually increasing with the increase in aldehyde excess. However, the extent of modification was almost reaching a plateau when aldehyde to polymer ratio exceeded the value of 5.63:1.

Based on a detailed analysis of all the experiments in Table S4 and Figures S42-S43, it can be concluded that the optimal reaction conditions for the modification of **PHU_1_** with butyraldehyde are as follows: temperature – 70°C, time – 24 h, *p*-TSA to polymer ratio – 0.38:1 and butyraldehyde to polymer ratio – 5.63:1.

**Figure S45.** Modification degree in **PHU_1_-Bu** vs temperature (**A**), reaction duration (**B**), catalyst to polymer ratio (**C**) and aldehyde to polymer ratio (**D**).

**Figure S46.** Dependence of **PHU_1_-Bu** modification degree on catalyst type.

Table S4. Modification of PHU_1_ with butyraldehyde (PHU_1_-Bu).

| **Optimization parameter** | **Entry** | **Time, h** | **T,**  **°C** | **[pTSA]: [polymer]** | **[butyraldehyde]: [polymer]** | **Yield, %^a^** | **Mod. Degree, %^b^** |
| --- | --- | --- | --- | --- | --- | --- | --- |
| Temperature | 1 | 24 | 40 | 0.38 | 7 | 78 | 55 |
|  | 2 | 24 | 50 | 0.38 | 7 | 71 | 77 |
|  | 3 | 24 | 60 | 0.38 | 7 | 66 | 78 |
|  | 4 | 24 | 65 | 0.38 | 7 | 67 | 82 |
|  | 5 | 24 | 70 | 0.38 | 7 | 70 | 85 |
|  | 6 | 24 | 75 | 0.38 | 7 | 67 | 84 |
|  | 7 | 24 | 80 | 0.38 | 7 | 71 | 82 |
|  | 8 | 24 | 90 | 0.38 | 7 | 73 | 80 |
| Time | 9 | 24 | 40 | 0.38 | 7 | 78 | 55 |
|  | 10 | 36 | 40 | 0.38 | 7 | 77 | 59 |
|  | 11 | 48 | 40 | 0.38 | 7 | 78 | 72 |
|  | 12 | 60 | 40 | 0.38 | 7 | 70 | 88 |
|  | 13 | 72 | 40 | 0.38 | 7 | 74 | 90 |
| [pTSA]:[polymer] | 14^c^ | 72 | 70 | 0.09 | 7 | 61 | 74 |
|  | 15 | 72 | 70 | 0.17 | 7 | 51 | 83 |
|  | 16 | 72 | 70 | 0.22 | 7 | 66 | 87 |
|  | 17 | 72 | 70 | 0.28 | 7 | 58 | 82 |
|  | 18 | 72 | 70 | 0.38 | 7 | 54 | 81 |
|  | 19 | 72 | 70 | 0.56 | 7 | 55 | 82 |
|  | 20 | 72 | 70 | 1.13 | 7 | 50 | 77 |
| [butyraldehyde]: [polymer] | 21 | 72 | 70 | 0.38 | 2 | 40 | 68 |
|  | 22 | 72 | 70 | 0.38 | 3 | 55 | 74 |
|  | 23 | 72 | 70 | 0.38 | 4 | 38 | 80 |
|  | 24 | 72 | 70 | 0.38 | 5.63 | 30 | 83 |
|  | 25 | 72 | 70 | 0.38 | 7 | 54 | 84 |
|  | 26 | 72 | 70 | 0.38 | 9 | 49 | 85 |

^a^ Isolated yield; ^b^ Calculated using ^1^H NMR (see section V.3.1.); ^c^ Partially cross-linked.

# VIII. DSC plots of polymers.

**Figure S47.** DSC plot of **PHU_1_** at a heating rate of 5°C/min.

**Figure S48.** DSC plot of **PHU_1_** at a heating rate of 10°C/min.

**Figure S49.** DSC plot of **PHU_2_** at a heating rate of 10°C/min.

**Figure S50.** DSC plot of **PU** at a heating rate of 5°C/min.

**Figure S51.** DSC plot of **PU** at a heating rate of 10°C/min.

**Figure S52.** DSC plot of **PHU_1_-Bu** at a heating rate of 5°C/min.

**Figure S53.** DSC plot of **PHU_1_-Bu** at a heating rate of 10°C/min.

**Figure S54.** DSC plot of **PHU_1_-Hept** at a heating rate of 5°C/min.

**Figure S55.** DSC plot of **PHU_1_-Hept** at a heating rate of 10°C/min.

**Figure S56.** DSC plot of **PHU_1_-FHept** at a heating rate of 5°C/min.

**Figure S57.** DSC plot of **PHU_1_-FHept** at a heating rate of 10°C/min.

**Figure S58.** DSC plot of **PHU_1_-Ph** at a heating rate of 5°C/min.

**Figure S59.** DSC plot of **PHU_1_-Ph** at a heating rate of 10°C/min.

**Figure S60.** DSC plot of **PHU_1_-ArCN** at a heating rate of 5°C/min.

**Figure S61.** DSC plot of **PHU_1_-ArCN** at a heating rate of 10°C/min.

**Figure S62.** DSC plot of **PHU_1_-ArCF_3_** at a heating rate of 5°C/min.

**Figure S63.** DSC plot of **PHU_1_-ArCF_3_** at a heating rate of 10°C/min.

**Figure S64.** DSC plot of **PHU_1_-PrPh** at a heating rate of 5°C/min.

**Figure S65.** DSC plot of **PHU_1_-PrPh** at a heating rate of 10°C/min.

**Figure S66.** DSC plot of **PHU_2_-PrPh** at a heating rate of 10°C/min.

# IX. DMTA plots of polymers.

**Figure S67.** Storage modulus (E’) and tan δ plots for **PHU_1_**, **PU** and modified **PHU_1_** polymers.

# X. Representative WAXD plots for qualitative analysis

**Figure S68.** WAXD plot of **PHU_1_**.

**Figure S69.** WAXD plot of **PHU_1_-ArCN**.

**Figure S70.** WAXD plot of **PHU_1_-Ph**.

**Figure S71.** WAXD plot of control **PU**.

# XI. Water adsorption.

**Figure S72.** Water adsorption in **PHU_1_**, **PU** and modified **PHU_1_** polymers at 45, 65 and 85% RH.

**Figure S73.** Water adsorption in **PHU_2_** and modified **PHU_1_-PrPh** polymers at 45, 65 and 85% RH.

# XII. Storage moduli (G’) of polymers at different humidity levels (via torsional rheometry).

**Figure S74.** Frequency dependence of storage moduli (G’) for **PHU_1_**, **PU** and modified **PHU_1_** polymers measured via torsion rheometry at 25°C after conditioning for 3 days at 45, 65 and 85% RH.

**Figure S75.** Frequency dependence of storage moduli (G’) for **PHU_2_** and **PHU_2_-PrPh** measured via torsion rheometry at 25°C after conditioning for 3 days at 45, 65 and 85% RH.

**Figure S76**. Frequency dependence of storage moduli (G’) for **PHU_1_-PrPh** measured via torsion rheometry at 25°C after conditioning for 3 and 14 days at 65 and 85% RH.

**Table S5.** Changes in T_α_ values of **PU** and PHU samples at different levels of water uptake.

| Sample | Mod. degree | ***Dry*** | ***45% RH*** | | ***65% RH*** | | ***85% RH*** | |
| --- | --- | --- | --- | --- | --- | --- | --- | --- |
|  |  | T_α_ (°C)^a^ | Water (w/w)^b^ | T_α_ (°C)^c^ | Water (w/w)^b^ | T_α_ (°C)^c^ | Water (w/w)^b^ | T_α_ (°C)^c^ |
| **PU** | - | 42 | 0.4% | 40 | 0.4% | 40 | 1.1% | 38 |
| **PHU_1_** | - | 81 | 2.0% | 70 | 3.7% | 61 | 7.5% | 43 |
| **PHU_1_-Bu** | 84% | 56 | 1.1% | 51 | 2.1% | 46 | 3.6% | 40 |
| **PHU_1_-Ph** | 43% | 86 | 0.7% | 82 | 1.7% | 76 | 3.7% | 65 |
| **PHU_1_-PrPh** | 80% | 51 | 0.2% | 50 | 1.1% | 46 | 2.4% | 41 |
| **PHU_1_-ArCF_3_** | 61% | 71 | 0.8% | 67 | 1.5% | 63 | 3.2% | 55 |

^a^ T_α_ (taken as peak maximum from the loss modulus (E”) curve) measured by DMTA at a heating rate of 5 °C/min; ^b^ Determined after 11 days of conditioning at selected humidity levels and 22 °C; ^c^ Calculated using the Fox equation^[15]^: $\frac{1}{T_{\alpha}}= \frac{w_{1}}{T_{\alpha1}}+\frac{w_{2}}{T_{\alpha2}}$, where in this case T_α1_, T_α2_, w_1_ and w_2_ are the main relaxation temperatures and weight fractions of polymer and water, respectively, and where T_α_ for water is taken as 136 K^[16]^.

# XIII. ^1^H NMR spectra and GPC plots of selected modified PHUs

**Figure S77**. Comparison of ^1^H NMR spectra of (A) pristine **PHU_1_-Hept** and (B) **PHU_1_-Hept** after exposure to 85% RH for 4 weeks.

**Figure S78**. Comparison of ^1^H NMR spectra of (A) pristine **PHU_1_-Ph** and (B) **PHU_1_-Ph** after exposure to 85% RH for 4 weeks.

**Figure S79**. Comparison of ^1^H NMR spectra of (A) pristine **PHU_1_-ArCF_3_** and (B) **PHU_1_-ArCF_3_** after exposure to 85% RH for 4 weeks.

**Figure S80**. GPC plots of pristine (green) and conditioned for 4 weeks at 85% RH (red) modified PHUs: (A) **PHU_1_-Hept**, (B) **PHU_1_-Ph**, (C) **PHU_1_-ArCF_3_**.

**Figure S81**. GPC plots of **PHU_1_** (red), **PHU_1_-Hept** (green) and **PHU_1_-ArCN** (blue).

# XIV. Tensile testing

**Table S6**. Tensile strength, elongation at break (ε) and tensile modulus of **PHU_1_**, **PHU_1_-Bu** and **PHU_1_-CF_3_.**

| Sample | Mod. degree | Tensile strength (MPa) | ε (%) | Young’s modulus (MPa) |
| --- | --- | --- | --- | --- |
| PHU_1_ | - | 20.7±1.1 | 1.77±0.26 | 1580±45 |
| PHU_1_-Bu | 84% | 10.2±1.8 | 1.79±0.19 | 880±80 |
| PHU_1_-ArCF_3_ | 61% | 6.7±0.7 | 0.80±0.09 | 1060±125 |

**Figure S82**. Tensile stress vs tensile strain plots for (A) **PHU_1_**, (B) **PHU_1_-Bu** and (C) **PHU_1_-CF_3_.**

1. Fulmer, G. R.; Miller, A. J. M.; Sherden, N. H.; Gottlieb, H. E.; Nudelman, A.; Stoltz, B. M.; Bercaw, J. E.; Goldberg, K. I. *Organometallics* **2010**, *29* (9), 2176–2179. <https://doi.org/10.1021/om100106e>.
2. Kajiyama, T.; MacKnight, W. J. *Polym. J.* **1970**, 1 (5), 548–554. <https://doi.org/10.1295/polymj.1.548>.
3. (a) Rieger, J. *Polym. Test.* **2001**, *20* (2), 199–204. <https://doi.org/10.1016/S0142-9418(00)00023-4>; (b) Lei, Z.; Xing, W.; Wu, J.; Huang, G.; Wang, X.; Zhao, L. *J. Therm. Anal. Calorim.* **2014**, *116* (1), 447–453. <https://doi.org/10.1007/s10973-013-3526-0>.
4. Schmidt, S.; Gatti, F. J.; Luitz, M.; Ritter, B. S.; Bruchmann, B.; Mülhaupt, R. Erythritol Dicarbonate as Intermediate for Solvent- and Isocyanate-Free Tailoring of Bio-Based Polyhydroxyurethane Thermoplastics and Thermoplastic Elastomers. *Macromolecules* **2017**, *50* (6), 2296–2303. <https://doi.org/10.1021/acs.macromol.6b02787> .
5. Salvado, V.; Dolatkhani, M.; Grau, É.; Vidil, T.; Cramail, H. Sequence-Controlled Polyhydroxyurethanes with Tunable Regioregularity Obtained from Sugar-Based Vicinal Bis-Cyclic Carbonates. *Macromolecules* **2022**, *55* (16), 7249–7264. https://doi.org/10.1021/acs.macromol.2c01112.
6. Magliozzi, F.; Chollet, G.; Grau, E.; Cramail, H. *ACS Sustain. Chem. Eng.* **2019**, *7*, 17282–17292. <https://doi.org/10.1021/acssuschemeng.9b04098>.
7. Kasmi, N., Pinel, C., da Silva Perez, D., Dieden, R., Habibi, Y. Polymer Chemistry, **2021**, 12(7), 991–1001. <https://doi.org/10.1039/d0py01512a>
8. <https://www.sigmaaldrich.com/deepweb/assets/sigmaaldrich/quality/spectra/193/748/FNMR010706.pdf>. Retrieved on 20.04.2023.
9. (a) Kajiyama, T.; MacKnight, W. J. *Polym. J.* **1970**, 1 (5), 548–554. <https://doi.org/10.1295/polymj.1.548>; (b) Li, Y.; Ren, Z.; Zhao, M.; Yang, H.; Chu, B. *Macromolecules* **1993**, *26* (4), 612–622. <https://doi.org/10.1021/ma00056a010>; (c) Smirnova, N. N.; Kandeev, K. V; Bykova, T. A.; Kulagina, T. G.; Fainleib, A. M. *Russ. Chem. Bull.* **2006**, *55* (5), 845–851. <https://doi.org/10.1007/s11172-006-0341-9>.
10. (a) Blain, M.; Cornille, A.; Boutevin, B.; Auvergne, R.; Benazet, D.; Andrioletti, B.; Caillol, S. Hydrogen Bonds Prevent Obtaining High Molar Mass PHUs. *J. Appl. Polym. Sci.* **2017**, *134* (45), 44958. <https://doi.org/10.1002/app.44958>;
    (b) Besse, V.; Camara, F.; Méchin, F.; Fleury, E.; Caillol, S.; Pascault, J.; Boutevin, B. How to Explain Low Molar Masses in PolyHydroxyUrethanes ( PHUs ). *Eur. Polym. J.* **2015**, *71*, 1–11. <https://doi.org/10.1016/j.eurpolymj.2015.07.020>;
    (c) Magliozzi, F.; Chollet, G.; Grau, E.; Cramail, H. Benefit of the Reactive Extrusion in the Course of Polyhydroxyurethanes Synthesis by Aminolysis of Cyclic Carbonates. *ACS Sustain. Chem. Eng.* **2019**, *7*, 17282–17292. <https://doi.org/10.1021/acssuschemeng.9b04098>
11. (a) Golovanov, D. G.; Lyssenko, K. A.; Antipin, M. Y.; Vygodskii, Y. S.; Lozinskaya, E. I.; Shaplov, A. S. *Cryst. Growth Des.* **2005**, *5* (1), 337–340. <https://doi.org/10.1021/cg030086i>; (b) Golovanov, D. G.; Lyssenko, K. A.; Vygodskii, Y. S.; Lozinskaya, E. I.; Shaplov, A. S.; Antipin, M. Y. *Russ. Chem. Bull.* **2006**, *55* (11), 1989–1999. <https://doi.org/10.1007/s11172-006-0541-3>.
12. (a) Somers, A.; Howlett, P.; MacFarlane, D.; Forsyth, M. *Lubricants* **2013**, *1* (1), 3–21. <https://doi.org/10.3390/lubricants1010003>; (b) Zhou, Y.; Qu, J. *ACS Appl. Mater. Interfaces* **2017**, *9* (4), 3209–3222. <https://doi.org/10.1021/acsami.6b12489>.
13. Fernández, M. D.; Fernández, M. J.; Hoces, P. *J. Appl. Polym. Sci.* **2006**, *102* (5), 5007–5017. <https://doi.org/10.1002/app.25004>.
14. Rumyantsev, M.; Rumyantsev, S.; Kazantsev, O. A.; Kamorina, S. I.; Korablev, I. A.; Kalagaev, I. Y. Managing of Hydrogen Bonding in Aqueous Poly(Vinyl Alcohol) Solutions: New Perspectives towards Preparation of More Homogeneous Poly(Vinyl Butyral). *J. Polym. Res.* **2020**, *27* (3), 52. <https://doi.org/10.1007/s10965-020-2031-y>.
15. Fox, T. G. Influence of Diluent and of Copolymer Composition on the Glass Temperature of a Polymer System. *Bull. Am. Phys. Soc.* **1956**, *1*, 123.
16. Capaccioli, S.; Ngai, K. L. Resolving the Controversy on the Glass Transition Temperature of Water? *J. Chem. Phys.* **2011**, *135* (10). <https://doi.org/10.1063/1.3633242>.
